# Defining pediatric polypharmacy: A scoping review

**DOI:** 10.1371/journal.pone.0208047

**Published:** 2018-11-29

**Authors:** Paul M. Bakaki, Alexis Horace, Neal Dawson, Almut Winterstein, Jennifer Waldron, Jennifer Staley, Elia M. Pestana Knight, Sharon B. Meropol, Rujia Liu, Hannah Johnson, Negar Golchin, James A. Feinstein, Shari D. Bolen, Lawrence C. Kleinman

**Affiliations:** 1 Department of Population & Quantitative Health Sciences, Case Western Reserve University, Cleveland, Ohio, United States of America; 2 Department of Clinical Sciences, University of Louisiana at Monroe College of Pharmacy, Monroe, Louisiana, United States of America; 3 Department of Medicine, MetroHealth Medical Center, Cleveland, Ohio, United States of America; 4 Center for Health Care Research and Policy, MetroHealth, Cleveland, Ohio, United States of America; 5 Department of Pharmaceutical Outcomes and Policy, University of Florida, Gainesville, Florida, United States of America; 6 Department of Epidemiology & Biostatistics, University of Florida, Gainesville, Florida, United States of America; 7 Division of Pediatric Neurology and Epilepsy, Rainbow Babies and Children’s Hospital, University Hospitals, Cleveland, Ohio, United States of America; 8 School of Medicine, Case Western Reserve University, Cleveland, Ohio, United States of America; 9 Rainbow Babies and Children’s Hospital, University Hospitals, Cleveland, Ohio, United States of America; 10 Epilepsy Center/ Neurological Institute, Cleveland Clinic, Cleveland, Ohio, United States of America; 11 UH Rainbow Center for Child Health and Policy, University Hospitals, Cleveland, Ohio, United States of America; 12 School of Pharmacy, University of Washington, Seattle, Washington, United States of America; 13 Department of Pediatrics, University of Colorado School of Medicine, Aurora, Colorado, United States of America; Wayne State University, UNITED STATES

## Abstract

**Objectives:**

Lack of consensus regarding the semantics and definitions of pediatric polypharmacy challenges researchers and clinicians alike. We conducted a scoping review to describe definitions and terminology of pediatric polypharmacy.

**Methods:**

Medline, PubMed, EMBASE, CINAHL, PsycINFO, Cochrane CENTRAL, and the Web of Science Core Collection databases were searched for English language articles with the concepts of “polypharmacy” and “children”. Data were extracted about study characteristics, polypharmacy terms and definitions from qualifying studies, and were synthesized by disease conditions.

**Results:**

Out of 4,398 titles, we included 363 studies: 324 (89%) provided numeric definitions, 131 (36%) specified duration of polypharmacy, and 162 (45%) explicitly defined it. Over 81% (n = 295) of the studies defined polypharmacy as two or more medications or therapeutic classes. The most common comprehensive definitions of pediatric polypharmacy included: two or more concurrent medications for ≥1 day (n = 41), two or more concurrent medications for ≥31 days (n = 15), and two or more sequential medications over one year (n = 12). Commonly used terms included polypharmacy, polytherapy, combination pharmacotherapy, average number, and concomitant medications. The term polypharmacy was more common in psychiatry literature while epilepsy literature favored the term polytherapy.

**Conclusions:**

Two or more concurrent medications, without duration, for ≥1 day, ≥31 days, or sequentially for one year were the most common definitions of pediatric polypharmacy. We recommend that pediatric polypharmacy studies specify the number of medications or therapeutic classes, if they are concurrent or sequential, and the duration of medications. We propose defining pediatric polypharmacy as “the prescription or consumption of two or more distinct medications for at least one day”. The term “polypharmacy” should be included among key words and definitions in manuscripts.

## Introduction

Polypharmacy is typically referred to as the concurrent use of multiple medications by an individual [1]. While widely recognized as a problem in the elderly population, polypharmacy is increasingly acknowledged as a common concern in pediatric patients [2–8] with both potential benefits such as control of complex or multiple disease conditions [9–12] and harms such as adverse drug effects, drug-to-drug interaction, hospitalization, poor medication adherence, mortality, resource wastage, burden of medical care, and high cost of healthcare [13–18]. Despite the increasing use of polypharmacy in children, there is not yet a uniform definition of polypharmacy in pediatric patients [19–21]. Factors such as number and duration of medications, medication classes, appropriateness of medications, medical conditions, and clinical setting are usually considered when defining polypharmacy, resulting in a variety definitions.

Definition variations pose challenges for researchers and clinicians. For example, if there are three pediatric patients, one an asthmatic child who is taking three essential medications, the next with multiple disease conditions that is taking 5 medications, and the last with ADHD taking two stimulants; are all these three children receiving polypharmacy? In adults, the term polypharmacy is typically used to refer to concurrent use of five or more medications [22–26], and comorbidities are the major influence for polypharmacy. In contrast, polypharmacy in pediatrics typically represents as few as two concurrent medications [27–34] prescribed for a single disease [35–39]. There is no contextual definition of polypharmacy that quantifies the magnitude, duration of exposure, and clinical implications of pediatric polypharmacy. This paper examines how the literature uses the term polypharmacy, along with related terminology, when considering the use of medications in children. It is important to have a consistent way of defining pediatric polypharmacy that will enable comparisons across study populations and standardization of research methods in the field.

The term polypharmacy first appeared in the medical literature over 150 years ago when it referred to multiple-ingredient preparations [1,40]. However, it was first introduced in the Medical Subject Headings (MeSH), the controlled vocabulary thesaurus used for indexing articles in the National Library of Medicine’s Medline database, in 1997 [41]. Since then, this term has been used in the literature with different meanings and definitions [2,4,5,7,11,13,14,16,18]. Adding to the conundrum, other terms such as *polytherapy*, *multi-drug therapy*, *multiple pharmacotherapy*, and *average number of medications* are oftentimes used to denote polypharmacy.

Another issue regarding pediatric polypharmacy, along with the number of medications, includes whether the medications overlap, and if they do, the duration of medication overlap. Definitions also vary on whether polypharmacy is assessed within or between medication classes, the clinical setting where polypharmacy is assessed, and the type and number of disease conditions in which polypharmacy is assessed. A recent systematic review by Masnoon, for example, identified 110 adult studies about polypharmacy that included 138 unique definitions of polypharmacy and associated terms [22]. Studies that stated numerical definitions (n = 51) predominantly reported polypharmacy as five or more medications. The number of medications constituting polypharmacy in Mansoon’s review ranged from two or more to 21 or more, and the most frequent period defining polypharmacy was 90 or more days with a range from one or more days to 240 or more days. Thus far, neither a common and consistent number of medications, nor a period of overlap for pediatric polypharmacy has been established.

We illustrate the disparity in defining pediatric polypharmacy and quantifying related exposures and outcomes with three studies. Working with all generic medications in Medicaid insurance claims, Feinstein et al.[42] graded pediatric outpatient polypharmacy exposure as low (2–5 medications), medium (5–9) and high (≥10) medication count (depth), and low (1–30 days) and high (≥31) concurrent medication duration. Similarly, Chen et al.[19] defined outpatient pediatric psychotropic polypharmacy as ≥ 2 concurrent and non-concurrent medications at different duration cut offs (≥14, ≥30, ≥60, and ≥90 days). Without defining it, Feudtner et al.[43] quantified exposure to inpatient polypharmacy by computing daily and cumulative number of medications during hospital stay, which was 3–9 medications per day and 21–42 medications per extended hospital stay, respectively. These numbers varied by age, hospital type, and disease condition. The prevalence of pediatric polypharmacy within and across these studies ranged from 18% to 100%. The intensity of exposure to polypharmacy is difficult to quantify.

Therefore, we sought to examine the definition of pediatric polypharmacy by conducting a scoping review of the literature. Our scoping review sought answers to the following questions. First, what definitions and descriptions are used for pediatric polypharmacy? Second, which definitions of pediatric polypharmacy are most frequent, comprehensive, or applicable to different types of research questions? We then use this information to offer suggestions regarding pediatric polypharmacy research and practice. This report follows the PRISMA 2009 checklist (S1 Checklist).

## Methods

Our scoping review followed the methodological framework proposed by Arksey and O’Malley, and enhanced by others [44–50]. A scoping review is a form of knowledge synthesis that addresses an exploratory research question aimed at mapping key concepts, types of evidence, and gaps in research related to a defined area or field by systematically searching, selecting, and synthesizing existing knowledge [51]. The methodology included articulating the research question; identifying relevant studies; selecting qualifying studies; extracting relevant information; collating and summarizing the information; and consulting experts in the field. Our detailed methodology is available in the protocol at the journal website (S1 File) and in our methods manuscript[52]. We briefly outline the methods below.

### Identification of relevant studies

A search strategy including both free text and controlled vocabulary for the concepts of “polypharmacy” and “children” was applied to eight bibliographic databases from inception to October 2016 and was updated on July 11, 2017. The databases included: Ovid Medline, PubMed, EMBASE, Ebsco CINAHL, Ovid PsycINFO, Cochrane CENTRAL, ProQuest Dissertations & Theses A&I, and the Web of Science Core Collection. Our medical librarian developed and applied the search criteria to the databases. Search queries for each database are available at the journal website (S2 File). Additionally, we conducted a hand search of the bibliographies of six relevant review articles and thirty randomly selected included studies.

### Inclusion and exclusion criteria

Studies that defined or assessed polypharmacy in children as an aim, outcome, predictor, or covariate were included. We excluded reviews, clinical trials, case series, case reports, conference abstracts, letters, comments, and opinion pieces, studies on polypharmacy in pregnancy, those related to breast milk, those that did not differentiate between children and adults, or those that were not in English. Consistent with scoping review methodology, we did not assess quality of included studies [44]. We excluded clinical trials from this epidemiological study of pediatric polypharmacy because of their methodological uniqueness.

### Data extraction

Like the screening forms, the data extraction form was developed iteratively and piloted on 100 titles and abstracts with modifications prior to use in the full scoping review. We extracted information regarding: 1) study characteristics including design, data sources, years, country, and clinical setting where the study was conducted; 2) disease conditions; 3) medications and their therapeutic classes; and 4) definitions and terminology of polypharmacy including number of medications, concurrence of medications, and text descriptions. The therapeutic classification was adapted from the American Hospital Formulary[53], with modifications informed by findings from the pilot phase, which guided the pre-coded part of the extraction form. In this manuscript, we refer to higher level classes as categories to differentiate them from lower level classes used as units of measures of polypharmacy in certain studies. We extracted information about the number of medications defining polypharmacy from any part of the manuscript including the tables and text, in addition to copying pieces of texts explicitly defining polypharmacy and pasting them in the extraction form. We then extracted detailed information on whether polypharmacy definition was considered at drug or class level, concurrent or sequential, period of concurrency, and any special additional characterization of polypharmacy. Concurrent polypharmacy referred to multiple medications issued or administered at the same time while sequential polypharmacy referred to multiple medications issued or administered during a specified period, although not necessarily concurrently.

Each article was independently reviewed for inclusion by two team members using standardized title/abstract and full text screening forms. Disagreements were resolved by consensus and were referred to the larger reviewer team during weekly meetings if paired reviewers were unable to resolve them. Data extraction was performed by one team member and reviewed by another team member for accuracy.

### Data analysis

We regarded a definition comprehensive if it included both a threshold (cut off) number of medications and an overlap or sequential period. We arranged polypharmacy descriptions, definitions, and terms by study characteristics, pharmacological categories, disease conditions, and research questions. When a study used more than one threshold for medication count or duration, we used the smallest threshold to ensure mutually exclusive categories.

We used Clarivate Analytics EndNote (X7) to organize and de-duplicate studies. EPPI Reviewer 4[54] was used for creating screening and extraction forms, assigning studies to reviewers, double screening studies, reconciling differences, cleaning data, and generating reports. The reports were exported to SAS 9.4 (SAS Institute, Cary North Carolina) for further data management, cleaning, and analysis.

## Results

### Summary of included studies

**Fig 1:**
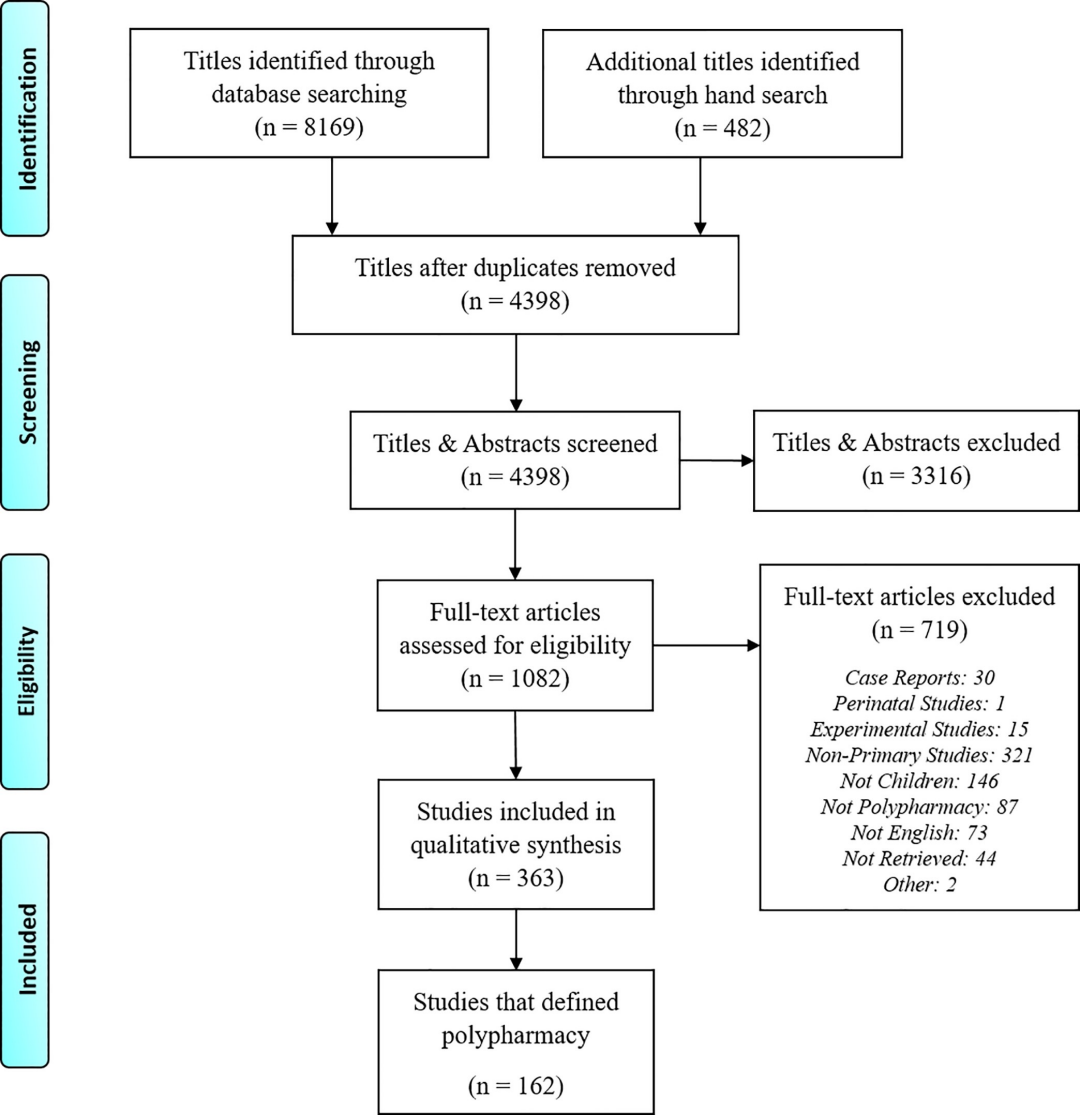
Flow Diagram of studies identified, screened, and extracted, PRISMA 2009.

Our database searches yielded 8,169 titles while the hand search yielded 482 titles. After de-duplication, 4,398 titles and abstracts were screened. From these, 1,082 qualifying studies were screened on full text, resulting in 363 studies that were reviewed (Fig 1).

Table 1 shows the characteristics of included studies and whether they explicitly defined polypharmacy. About 46% (n = 168) of the studies were published in 2011 or later, 65% (n = 235) were cross-sectional, and 34% (n = 124) were cohort studies. Primary data collection (47%, n = 172) and chart review (25%, n = 90) were the most common data sources. The most common study setting was outpatient of any kind: specialist, primary care, office, hospital, emergency room, or academic centers (54%, n = 197).

**Table 1:** Characteristics of included studies and the proportions that reported explicit definitions of polypharmacy.

Characteristic		Overall n (column %)	Defined Polypharmacy n (row %)
**All**	All	363 (100)	162 (44.6)
**Year of Publication**	Up to 2000	63 (17.4)	27 (42.9)
	2001–2010	132 (36.3)	57 (43.2)
	2011–2017	168 (46.3)	78 (46.4)
**Study Design**	Case Control	4 (1.1)	4 (100.0)
	Cross Sectional	235 (64.8)	102 (43.4)
	Prospective Cohort	56 (15.4)	23 (41.1)
	Retrospective Cohort	68 (18.7)	33 (48.5)
**Data Source**^a^	Primary Data Collection	172 (47.4)	72 (41.9)
	Chart Review	90 (24.8)	30 (33.3)
	Medical/Pharmacy Claims	44 (12.1)	32 (72.7)
	Electronic Records	17 (4.7)	11 (64.7)
	Drug Registry	15 (4.1)	10 (66.7)
	Others & Combinations	25 (6.9)	7 (28.0)
**Healthcare Setting**^c^	Outpatient	197 (54.3)	93 (47.2)
	Inpatient	77 (21.2)	23 (29.9)
	Inpatient & Outpatient	27 (7.4)	10 (37.0)
	Others	51 (14.1)	29 (56.9)
	Not reported	11 (3.0)	7 (63.6)
**Polypharmacy Role**^c^	Outcome	140 (38.6)	76 (54.3)
	Main Predictor	124 (34.1)	49 (39.5)
	Covariate	99 (27.3)	37 (37.4)
**Polypharmacy Level**^b^	Class	41 (11.3)	28 (68.3)
	Drug	299 (82.4)	121 (40.5)
	Combination	13 (6.3)	13 (8.0)
**Pharmacological Category**^a^	CNS Agents	169 (46.5)	67 (39.6)
	Psychotropic Agents	88 (24.2)	57 (64.8)
	Combination	99 (27.3)	34 (34.3)
	Not Reported	7 (2.0)	4 (57.1)
**Disease Condition**^b^	Psychiatric	67 (18.5)	39 (58.2)
	Somatic	33 (9.1)	9 (27.3)
	Epilepsy	150 (41.3)	58 (38.7)
	Combinations	53 (14.6)	33 (62.3)
	Not Reported	60 (16.5)	23 (38.3)

CNS = Central Nervous System.

Pearson X^2^ p-value comparing proportions of studies that defined polypharmacy between variable categories:

^a^ < .001

^b^ < .01

^c^ < .05.

Polypharmacy (prevalence) was the outcome of interest in 39% (n = 140), the main predictor in 34% (n = 124), and a covariate in 27% (n = 99) of studies. Other outcome measures or research questions included prognostic markers (26%, n = 93), adverse drug events (17%, n = 60), non-polypharmacy medication use (9%, n = 34), and drug monitoring (8%, n = 29) (S1 Table). The most frequent pharmacological categories were central nervous system agents (47%, n = 169) and psychotropic agents (24%, n = 88). Most studies evaluated polypharmacy in epilepsy (41%, n = 150) or psychiatric conditions (19%, n = 67).

About 45% (n = 162) of the studies specified a definition of polypharmacy in the text. Studies whose data sources were insurance claims, electronic health records, or drug registries were more likely to specify text definitions of polypharmacy (Table 1, p < .001). Other factors associated with offering a specific definition in the text were polypharmacy being an outcome (p < .05), polypharmacy assessment at therapeutic class level (p < .01), psychotropic medication polypharmacy (p < .001), and studies focused on psychiatric conditions (p < .01). Inpatient setting studies were less likely to define polypharmacy that studies conducted in other settings (p < .05).

### Semantics of polypharmacy

Table 2 shows polypharmacy terms by disease conditions. Studies of pediatric polypharmacy used a variety of terms to describe polypharmacy, including those that used compound words. We referred to similar compound words with one term. The most commonly used terms were “combination” (46%, n = 166), “polytherapy” (46%, n = 165), “polypharmacy” (40%, n = 144), “multiple” (37%, n = 133), and “average number” (34%, n = 124). “Average number” of medications was typically used as a term to describe polypharmacy, at an aggregate level, in situations where many medications were considered, particularly in inpatient settings or the treatment of multiple diseases. The term polytherapy was more frequently used in epilepsy studies than psychiatry studies (89% vs 6%, p < .001) while the term “polypharmacy” was more frequently used in psychiatry than epilepsy studies (70% vs 14%, p < .001). The proportion of studies using the terms “polypharmacy” and “average number” increased over time while that using “co-prescription/co-medication” decreased over time.

**Table 2:** Polypharmacy terms and descriptions.

Terms Denoting Polypharmacy (*Equivalent Terms*)	Overall N (%) N = 363	Epilepsy n (%) n = 150	Psychiatry n (%) n = 67	Other n (%) n = 146
**Add-on**	45 (12.4)	19 (12.7)	10 (14.9)	16 (11.0)
**Adjunctive**	39 (10.7)	14 (9.3)	13 (19.4)	12 (8.2)
**Augment**	24 (6.6)	0 (0.0)	14 (20.9)	10 (6.9)
**Average Number** *of Medications*, *(Prescriptions*, *Drugs)*	124 (34.2)	36 (24.0)	15 (22.4)	73 (50.0)
**Combination** *Pharmacotherapy (Therapy*, *Products*, *Medication*, *Treatment)*	166 (45.7)	61 (40.7)	44 (65.7)	61 (41.8)
**Concomitant** *Medications (Drugs*, *Drug Use*, *Classes*, *Therapy*, *Treatment*, *Regimen*, *Antiepileptic Drugs*, *Psychotropics)*	95 (26.2)	24 (16.0)	27 (40.3)	44 (30.1)
**Concurrent** *Medications (Therapy*, *Psychotropics)*	76 (20.9)	15 (19.7)	29 (38.2)	32 (42.1)
**Comedication** *(Coprescription*, *Cotreatment*, *Cotherapy*, *Copharmacy*, *Coadministration)*	48 (13.2)	19 (12.7)	10 (14.9)	19 (13.0)
**Dual** *(Di*, *Double) Therap****y***	6 (1.7)	5 (3.3)	0 (0.0)	1 (0.7)
**Multiple** *Medications (Drugs*, *Classes*, *Agents*, *Antiepileptic Drugs*, *Antipsychotics*, *Psychotropics)*	133 (36.6)	25 (16.7)	42 (62.7)	66 (45.2)
**Polypharmacy**	144 (39.7)	21 (14.0)	47 (70.2)	76 (52.1)
**Polytherapy**	165 (45.5)	133 (88.7)	4 (6.0)	28 (19.2)
**Simultaneous**	26 (7.2)	8 (5.3)	9 (13.4)	9 (6.2)
**Number of Terms Per Study**				
1	62 (17.1)	33 (22.0)	4 (6.0)	25 (17.1)
2	102 (28.1)	52 (34.7)	9 (13.4)	41 (28.1)
3	81 (22.3)	40 (26.7)	13 (19.4)	28 (19.2)
4	56 (15.4)	13 (8.7)	18 (26.9)	25 (17.1)
5+	62 (17.1)	12 (8.0)	23 (34.3)	27 (18.5)

The “other” disease category includes 33 studies of somatic diseases (predominantly infections = 18, asthma = 7, other respiratory diseases = 6), 53 studies of combinations of epilepsy, psychiatry (predominantly bipolar disorder = 25, depression = 29, ADHD = 31, psychosis = 22, anxiety = 28, autism = 11, conduct order = 9), and/or somatic (Intellectual and Developmental Disability (IDD) = 36, asthma = 3, infections = 4) diseases, and 60 studies that did not report disease conditions.

**Fig 2:**
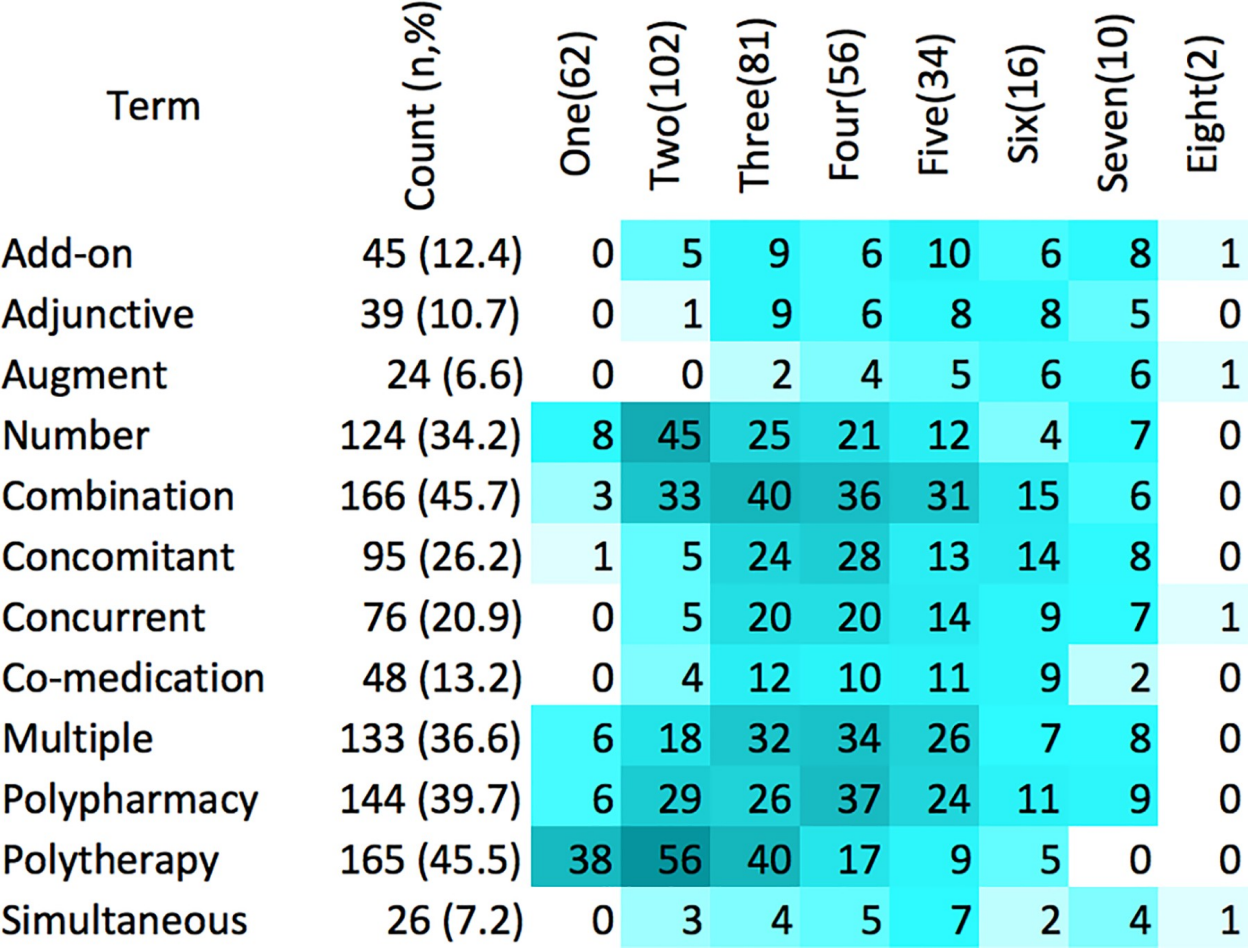
Heat map showing combinations of polypharmacy term. 1. The count column shows number (out of 363) and percent of studies that used a specified term. 2. Columns One(62) to Eight(2) specify number of studies with the respective exact number of terms. 3. Cells show number of studies with corresponding term in combination with other terms.

Different polypharmacy terms were used concurrently in the same manuscript. While most studies used one to three terms, there were studies which used more (Fig 2 and Table 2), suggesting that terms were at times used in a more technical or definitional manner, and at times as vernacular. This conclusion is supported by the observation that manuscripts that used multiple terms tended to have a predominant term that was used more frequently than other terms. The epilepsy literature predominantly used three or fewer polypharmacy terms while psychiatry literature predominantly used three or more terms. Twenty-eight studies used six or more polypharmacy terms concurrently (Fig 2). For example, one study [55] used polypharmacy, multiple medications, multiple drug claims, multiple refills, multiple medication claims, combination prescriptions, combination pharmacotherapy, concurrent treatment, and duplication medication claims.

The terms co-medication, co-prescription, and concomitant medications sometimes referred to additional medications when polypharmacy was assessed for a group of medications such as antipsychotics [56–59]. Add-on, adjunctive, and augment inherently referred to beneficial polypharmacy. Multiple, combination, and concurrent medications were frequently used as English words rather than reference to polypharmacy.

### Types of polypharmacy

Table 3 shows different aspects of defining polypharmacy, including level, period, and number of medications. About 82% (n = 299) of the studies assessed polypharmacy at the medication level, regardless of drug or therapeutic class while 18% (n = 64) of the studies assessed polypharmacy at the class level, regarding all medications from the same class as one unit. Studies focused on psychiatric medications were more likely to report polypharmacy by class than were studies of epilepsy (46% vs 0%, p < .001). Additionally, studies of class-level polypharmacy were more likely to report an explicit definition than those of drug-level polypharmacy (64% and 40% respectively, p < .01, data not shown).

**Table 3:** Definitions of pediatric polypharmacy, overall and by disease conditions.

Type of Definition		Overall (N = 363)	Epilepsy Only (n = 150)	Psychiatry Only (n = 67)	Other (n = 146)
**Polypharmacy Level**^a^	Class Level	64 (17.6)	0 (0.0)	31 (46.3)	33 (22.6)
	Drug Level	299 (82.4)	150 (100.0)	36 (53.7)	113 (77.4)
**Concurrent or** **Sequential**^a^	Concurrent	320 (88.2)	144 (96.0)	58 (86.6)	118 (80.8)
	Sequential Period	43 (11.9)	6 (4.0)	9 (13.4)	28 (19.2)
	≤1 Year	6 (14.0)	0 (0.0)	1 (11.1)	5 (17.9)
	1 Year	18 (41.9)	4 (66.7)	4 (44.4)	10 (35.7)
	≥2 Years	7 (16.3)	2 (33.3)	4 (44.4)	1 (3.6)
	Inpatient Period	12 (27.9)	0 (0.0)	0 (0.0)	12 (42.9)
**Number of Medications**^a^	≥2	295 (81.3)	141 (94.0)	56 (83.6)	98 (67.1)
	≥3	18 (5.0)	5 (3.3)	8 (11.9)	5 (3.4)
	≥4 /≥5	11 (3.0)	0 (0.0)	1 (1.5)	10 (6.9)
	Not Reported	39 (10.7)	4 (2.7)	2 (3.0)	33 (22.6)
**Number of Overlap Days**^a^	≥1	56 (15.4)	16 (10.7)	17 (25.4)	23 (15.8)
	≥31	18 (5.0)	5 (3.3)	9 (13.4)	4 (2.7)
	≥ 61	14 (3.9)	4 (2.7)	6 (9.0)	4 (2.7)
	Sequential	43 (11.9)	6 (4.0)	9 (13.4)	28 (19.2)
	Not reported	232 (63.9)	119 (79.3)	26 (38.8)	87 (59.6)

1. The “other” disease category includes 33 studies of somatic diseases (predominantly infections = 18, asthma = 7, other respiratory diseases = 6), 53 studies of combinations of epilepsy, psychiatry (predominantly bipolar disorder = 25, depression = 29, ADHD = 31, psychosis = 22, anxiety = 28, autism = 11, conduct order = 9), and/or somatic (Intellectual and Developmental Disability (IDD) = 36, asthma = 3, infections = 4) diseases, and 60 studies that did not report disease conditions.

2. The thresholds of >4 and ≥5 medications were combined because of small numbers.

3. Threshold number of days were collapsed as follows:

a. >1day category includes ≥1 day (51 studies) and ≥14 days (5)

b. >61 category includes ≥61 (5), ≥90 (3), ≥ 180 (4) and ≥365 (2)

c. Sequential includes ≥1 year (6), 1 year (18), 2 years (7), and hospital stay (12).

4. Pearson X^2^ p-value comparing proportions of studies by disease conditions and polypharmacy definition

^a^ < .001

^b^ < .01

^c^ < .05.

Eighty-eight percent (n = 320) of the studies assessed concurrent polypharmacy while 12% (n = 43) assessed sequential polypharmacy. Sequential polypharmacy was reported in 13% of psychiatry studies and only in 4% of epilepsy studies. Among the 43 studies that reported sequential polypharmacy, 42% (n = 18) considered an interval of one year, 28% (n = 12) used the hospital stay to define the relevant interval, 16% (n = 7) specified time intervals of longer than two years, and 14% (n = 6) specified intervals less than one year.

Regarding semantics, the terms “overlapping” [3,19,60] or “concomitant” polypharmacy [3,60,61] were used to indicate concurrence. Terms used to describe sequential polypharmacy included “temporal”, “cross-sectional”, “lifetime”, “non-overlapping”, or “non-concomitant” [61–63]. Other, less commonly used, language included “long-term” polypharmacy, which was defined variously as concurrence for more than 30, 60, or 180 days. “Excessive” polypharmacy referred to use of 5 or more and 10 or more concurrent medications. “Inappropriate” or “irrational” inflected a clinical judgment into the description of polypharmacy, suggesting use of unnecessary medications for a given individual. Three studies regarded “fixed-dose combinations”, meaning at least two medications combined in one pill/liquid preparation, as polypharmacy. Additional descriptors of polypharmacy included “depth” (numeric threshold), “therapeutic load” (numeric threshold), “cumulative exposure” (number of medications during hospitalization), “daily exposure” (number of medications per day of hospitalization), and “duplication” (same medication from different prescribers).

### Threshold number of medications and overlap periods

Eighty-nine percent (n = 324) of the studies used numeric definitions of polypharmacy either as part of the explicit text definition or in another section of the manuscript. Eighty-one percent (n = 295) of the studies used ≥2 medications as the threshold cut off for pediatric polypharmacy (Table 3). Studies of psychotropic medications were more likely to use a threshold of three or more medications than were studies of epilepsy (12% vs 3%, p < .001). The studies that we reviewed did not routinely report the length of overlap. Only 24% (n = 88) reported overlap periods: 15% (n = 56) reported ≥1 day, 5% (n = 18) reported >30 days, and 4% (n = 14) reported >60 days (Table 3). Nearly two-thirds (64%, n = 232) of the studies did not explicitly report the duration of concurrent medications.

### Comprehensive definition of polypharmacy

Table 4 shows combinations of medication count and duration thresholds that defined polypharmacy. Overall, only 30% (n = 108) of the studies specified both count and duration thresholds. The most frequent combinations were two or more medications for ≥1 days (11%, n = 41), two or more medications for ≥31days (4%, n = 15), and the sequential combination of two or more medications in one year (3%, n = 12). About 60% (n = 216) of the studies provided only a numeric threshold of medications and 6% (n = 23) of the studies provided only a duration threshold. The 16 studies that provided neither a numeric nor a duration threshold of polypharmacy were mainly conducted in the inpatient setting and defined polypharmacy as average number rather than a threshold number of medications. The average number of medications in these studies ranged from one to eighteen.

**Table 4:** Combinations of numeric and duration thresholds of medications defining pediatric polypharmacy.

Number of Medications	Duration in Days	Number of Studies	Percent
**At least 2 medications**	≥1 day	41	11.3
	≥31 days	15	4.1
	≥ 61 days	13	3.6
	Sequential	22	6.1
	Not reported	204	56.2
**At least 3 medications**	≥1 day	2	0.6
	≥31 days	3	0.8
	≥ 61 days	1	0.3
	Sequential	5	1.4
	Not reported	7	1.9
**At least 4/5 medications**	≥1 day	3	0.8
	Sequential	3	0.8
	Not reported	5	1.4
**Not reported**	≥1 day	10	2.8
	Sequential	13	3.6
	Not reported	16	4.4

1. To ensure mutually exclusive categories, the shortest threshold was presented when there were multiple medication thresholds or durations.

2. The thresholds of at least 4 and 5 medications were combined because of small numbers.

3. Threshold number of days were collapsed as follows:

a. >1day category includes ≥1 day (51 studies) and ≥14 days (5)

b. ≥61 category includes ≥61 (5), ≥90 (3), ≥ 180 (4) and ≥365 (2)

c. Sequential includes ≥1 year (6), 1 year (18), 2 years (7), and hospital stay (12).

d. Sequential includes ≥1 year (6), 1 year (18), 2 years (7), and hospital stay (12)

### Explicitly defining polypharmacy in the literature

We found 162 studies that offered 203 distinct definitions of polypharmacy in the text. These definitions were a subset of the overall definitions described above and had similar distributions of threshold number of medications or overlap periods. Only 19% (n = 35 outpatient, 3 inpatient) of the text definitions provided both a medication threshold number and a period (Table 5) [5,8,11,13,18,20,55,61,63–88]. A list of all 203 text definitions is available at the journal website (S2 Table). Studies with explicit text definitions were more likely to assess polypharmacy prevalence as the primary outcome measure (42% vs 23%, p < .001), and to mention side effects (29% vs 21%, p < .05) or drug-drug interactions (27% vs 16%, p = .057) as potential harms compared to those that did not have explicit definitions of polypharmacy. These findings were similar to those between studies with and without comprehensive definitions of polypharmacy. Of note, inpatient setting studies rarely reported explicit definitions of polypharmacy (Table 5).

**Table 5:** Studies that provided comprehensive explicit definitions of pediatric polypharmacy with number and duration of medications.

Author	Setting	Disease	Level	Number of Medications	Overlap Days	Explicit Definition
**Cho (2015)**	OP	Epilepsy	Drug	> = 2Meds	> = 1Day	• patients who received two or more AEDs on the same prescription date at least once
**Kanta (2014)**	OP	Epilepsy	Drug	> = 2Meds	NR	• two drugs were started simultaneously or second drug was added when first drug was not on maximum dose
**Carpay (1998)**	OP	Epilepsy	Drug	> = 2Meds	> = 30Days	• The concurrent use of 2 or more AEDs for more than 1 month
**Bhowmik (2013)**	OP	Psychiatry	Class	> = 2Meds	> = 1Day	• polytherapy was defined as receiving medications with minimum 1 day overlap between prescriptions from two or three different therapeutic classes within a specific month
**Gyllenberg (2012)**	OP	Psychiatry	Class	> = 2Meds	> = 1Day	• having purchased two psychotropic drugs from different drug classes during the same day.
**Dosreis (2011)**	OP	Psychiatry	Class	> = 2Meds	> = 30Days	• overlap of greater than or equal to 2 antipsychotics for more than 30 days.
**Logan (2015)**	OP	Psychiatry	Class	> = 2Meds	> = 30Days	• the simultaneous use of two or more different classes of psychotropic medication for a period of at least 30 consecutive days at any time during the 2 year study period for each child
**Spencer (2013)**	OP	Psychiatry	Class	> = 2Meds	> = 30Days	• polypharmacy was defined as at least 1 episode of multiclass polypharmacy. An episode of multiclass polypharmacy was defined as overlapping fills of medications across > = 2 classes for at least 30 days.
**Rushton (2001)**	OP	Psychiatry	Class	> = 2Meds	Sequential	• patients were described as combination prescription recipients if they received both a stimulant and an SSRI during the same calendar year.
**Mandell (2008)**	OP	Psychiatry	Class	> = 3Meds	> = 30Days	• concurrent use was coded when a child had prescriptions for > = 3 medications in different classes overlapping for at least 30 days.
**Rubin (2009)**	OP	Psychiatry	Class	> = 3Meds	> = 30Days	• concurrent use was coded when a child had prescriptions for > = 3 medications in different classes overlapping for at least 30 days.
**Rubin (2012)**	OP	Psychiatry	Class	> = 3Meds	> = 30Days	• concurrent use of 3 or more psychotropic medication classes for at least 30 days during the year
**Fontanella (2009)**	IP	Psychiatry	Class	> = 3Meds	NR	• the prescription of 3 or more medications from different drug classes at discharge
**Bali (2015)**	OP	Psychiatry	Drug	> = 2Meds	> = 14Days	• concomitant use of long acting stimulants and atypical antipsychotics was defined as receipt of both medications together for at least 14 days
**Kamble (2015)**	OP	Psychiatry	Drug	> = 2Meds	> = 14Days	• concurrent use or polypharmacy involving LAS and second-generation antipsychotics was defined as simultaneous receipt of both medications for at least 14 days
**Cornblatt (2007)**	OP	Psychiatry	Drug	> = 2Meds	> = 1Day	• 2 or more drugs taken at the same time
**Baeza (2014)**	OP	Psychiatry	Drug	> = 2Meds	> = 60Days	• defining polypharmacy as the receipt of 2 or more AP (antipsychotic) medications concurrently for more than 60 days, with no gaps of more than 15 days in the treatment
**Constantine (2010)**	OP	Psychiatry	Drug	> = 2Meds	> = 60Days	• antipsychotic polypharmacy was defined as the receipt of > = 2 antipsychotic medications concurrently for >60 days, with no gaps in polypharmacy treatment >15 days
**Lee (2016)**	OP	Psychiatry	Drug	> = 2Meds	> = 90Days	• concurrent use of 2 or more antipsychotics for 90 days
**Lee (2016)**	OP	Psychiatry	Drug	> = 3Meds	> = 60Days	• concurrent use of 3 or more antipsychotics for 60 days
**Lee (2016)**	OP	Psychiatry	Drug	> = 3Meds	> = 90Days	• concurrent use of 3 or more antipsychotics for 90 days
**Yoon (2012)**	OP	Somatic	Class	> = 2Meds	> = 1Day	• combination therapy was defined as prescription claims for 2 drug classes on the same or within 1 day.
**Jameel (2012)**	IP	Multiple	Class	> = 2Meds	> = 1Day	• polypharmacy: Nearly 20% of all the patients in our study were started on 2 or more psychotropic drugs simultaneously.
**dosReis (2005)**	OP	Multiple	Class	> = 2Meds	Sequential	• months of multiple use, which referred to the use of two or more different psychotropic classes within the same month
**Connor (1997)**	IP	Multiple	Drug	> = 2Meds	> = 1Day	• CPT (combined pharmacotherapy) was defined broadly as receiving two or more psychoactive agents at the same time
**Osunsanmi (2016)**	OP	Multiple	Drug	> = 2Meds	> = 180Days	• concurrent use of more than one ADHD medication for a continuous period of 6 months was referred to as cases on multiple medications.
**Schubart (2014)**	OP	Multiple	Both	> = 2Meds	> = 60Days	• concurrent use defined as use of two or more medications overlapping for at least 60 days
**Kalilani (2017)**	OP	Multiple	Both	> = 2Meds	> = 90Days	• polytherapy was defined as the prescription of lacosamide concomitantly with another AED(s) with an overlap of at least 90 days.
**Martin (2003)**	OP	Multiple	Both	> = 2Meds	Sequential	• multiple psychotropic pharmacotherapy was defined as having claims for prescriptions for medications in two or more different psychotropic drug classes during a seven-day period.
**Feinstein (2015)**	OP	NR	Drug	> = 2Meds	> = 1Day	• > = 2 concurrent medications for at least 1day
**Sharma (2016)**	OP	NR	Drug	> = 2Meds	NR	• the WHO standard for average number of drugs prescribed per patient encounter is 2.0. Rates higher than this standard are suggestive of polypharmacy.
**Feinstein (2015)**	OP	NR	Drug	> = 5Meds	> = 30Days	• depth and duration: The cut point for high-depth was > = 5 concurrent medications, the cut point for high-duration was > = 31 days
**Zoega (2009)**	OP	NR	Drug	> = 2Meds	> = 1Day	• concomitant drug use was defined as the dispensing of two or more different psychotropic chemical substances to a child on the same day at least once within the calendar year.
**Allaire (2016)**	OP	NR	Drug	> = 2Meds	> = 30Days	• having a prescription overlap of more than 30 days of two different second generation antipsychotics
**Hincapie-Castillo (2017)**	OP	NR	Drug	> = 2Meds	> = 30Days	• overlap of greater than 45 days in the active periods of two or more psychotropic medications with different active ingredients
**Hovstadius (2010)**	OP	NR	Drug	> = 5Meds	Sequential	• the prevalence of polypharmacy was defined as the proportion of individuals receiving five or more dispensed prescription drugs (DP> = 5) during a 3-month period.
**Hovstadius (2009)**	OP	NR	Drug	> = 5Meds	Sequential	• the prevalence of multiple medications was defined as the proportion of individuals who received five or more dispensed drugs during a 12-month period
**Hovstadius (2010)**	OP	NR	Drug	> = 10Meds	NR	• as a definition of excessive polypharmacy, we applied ten or more dispensed drugs (DP> = 10) for an individual during the study period

1. ADHD = Attention-deficit/hyperactivity disorder

2. AEDs = Antiepileptic drugs

3. DP = Dispensed prescription

4. IP = Inpatient

5. LAS = Long-acting stimulants

6. NR = Not Reported, mainly pharmacy based

7. OP = Outpatient

8. Sequential = Non-overlapping polypharmacy

9. SSRI = Selective serotonin reuptake inhibitor

10. WHO = World Health Organization

11. Multiple = More than one disease group, predominantly combination of epilepsy and psychiatry

## Discussion

A review of the literature to consider definitions and terms used in studies of pediatric polypharmacy was completed. The most common definition for polypharmacy in children included the use of two or more medications. Concurrent use of two or more medications was the exclusive definition in 60% of studies and was reported by 89% of the studies that we reviewed. We were surprised that less than half of the studies explicitly reported the minimum length of medication overlap. When overlap periods were reported, at least one or more days was the modal definition, with more than 30 days also used frequently. However, most of the studies that did not report overlap period implied at least one day, suggesting that researchers included overlap period in the definition to convey chronicity. Only 30% of the studies explicitly defined polypharmacy with both a threshold number of medications and an overlap or sequential period. Common terms that described use of multiple medications included polypharmacy, polytherapy, combination pharmacotherapy, multiple medications, and average number of medications. The lack of uniform definition or terminology of pediatric polypharmacy makes it difficult for health workers and researchers to assess and compare safety and efficacy of polypharmacy, which necessitates standardization of the definitions and terminology.

Our findings are similar to those of a systematic review conducted among adults that found 74% of the studies reviewed used medication count as the exclusive definition of polypharmacy[22]. Though, the threshold in that systematic review was five medications, compared to two medications in our study. The difference in threshold number of medications defining polypharmacy in children and adults is understandable, as children have less disease burden than do adults. This difference was illustrated by a population-based study that used a threshold of five medications for both adults and children and found that the prevalence of polypharmacy was 21.4% and 0.8%, respectively[18]. The small proportion of children with complex chronic conditions (CCC) are exceptional, as they tend to have a big burden of prescription medications [42,89]. A CCC is expected to last at least 12 months, and involves either several organs or one organ system severely enough to require specialty pediatric care and probably some period of hospitalization in a tertiary care center [90]. Another reason why pediatric polypharmacy definition has a lower threshold number of medications than adult polypharmacy is that most pediatric polypharmacy research has been driven by potential harm related to specific medications rather than medication burden or co-morbidity [35–39,91,92].

In our study, 17% of the text definitions were based on one or two index medications, and most definitions were based on medications for treating one disease condition. Only 15% of the studies evaluated polypharmacy without limiting it to particular medications or disease conditions. There is need to consider total real-world polypharmacy in addition to specific medications or disease conditions in order to evaluate harm related to various medication combinations, such as drug-drug interaction and non-adherence. A small proportion of studies qualified polypharmacy in terms of safety and magnitude by using descriptors such as appropriate/inappropriate, rational/irrational, excessive, or short-term/long-term polypharmacy. Widely using these terms would confer clinical meaning to the numeric or duration thresholds that are less meaningful.

Whether fixed-dose combinations can be considered polypharmacy was controversial in our research team. While the term polypharmacy was first coined to describe multi-ingredient preparations[40], contemporary researchers do not categorize fixed-dose combinations as polypharmacy[93–95]. This partially explains why disease conditions where fixed-dose combinations are used such as HIV, malaria, asthma, and hypertension were uncommon in our study. Fixed-dose combinations address problems of polypharmacy such as pill burden, drug-drug interactions, or dose-related side effects which would have inspired research questions.

The inpatient setting was underrepresented in our sample, making defining polypharmacy challenging using the conventional threshold number or duration of medications. Over 120 studies used average number of medications at admission or discharge from the inpatient facility as the measure of polypharmacy. Less than 10 studies referred to daily or cumulative average number of medications in order to quantify the large number of medications consumed by children during hospitalization [43,96]. However, average number of medications seems to build from the World Health Organization (WHO) guidelines, stating that an average number of medications per prescription or patient encounter greater than two is considered polypharmacy and is commonly used in international settings [84]. Inpatient polypharmacy should be characterized to capture its magnitude and associated risks over a short period of time. Neonatal intensive care units, where there is exposure to many medications over a long period, present even a greater challenge when characterizing inpatient pediatric polypharmacy[97–108].

Although thresholds of more than two medications or longer than 31 days of overlap were rare, they were more frequent in psychiatric studies than epilepsy studies. There have been more efforts to streamline psychotropic polypharmacy research than any other mediations or disease-related polypharmacy [6,19,109]. Moreover, psychiatry studies tended to operationalize their polypharmacy definition with threshold numbers or duration of medications, class or drug level in order to accommodate the inconsistencies in definitions [3,6,19,42,77,83].

Pediatric polypharmacy terms varied between epilepsy and psychiatry conditions—the two most frequent disease conditions where polypharmacy was evaluated. Epilepsy studies predominantly used the term polytherapy and hardly used other terms, while psychiatry and other somatic disease studies frequently used the term polypharmacy. However, several excluded psychiatry studies used the term polytherapy to refer to a combination of drug therapy and non-drug therapy, such as behavioral therapy. The predominance of the term “polytherapy” in epilepsy literature, coupled with the rarity of other terms, suggests consistency of terminology. However, readers who are used to the term “polypharmacy” are at a risk of missing literature that uses the term “polytherapy.” It is imperative that epilepsy researchers and providers build consensus with the rest of the research community on polypharmacy terminology to decrease inconsistencies in findings and improve the dissemination of knowledge.

The term polypharmacy has been used with negative connotation to imply harmful practices. In this regard, three essential medications for an asthmatic child or five medications for a child with multiple disease conditions might not be considered polypharmacy while two stimulants for ADHD may be considered polypharmacy because of their potential harm. The first two examples align within clinical guidelines while the third example may be outside clinical guidelines. Terms such as add-on, adjunctive, and augmentation, which seem to imply benefit, were often used. Clinical guidelines, harms, and benefits—including those resulting from combining medications—should be considered when defining pediatric polypharmacy.

When the outcomes or research questions of interest were related to the prevalence of polypharmacy or harm associated with medication use, the definition of polypharmacy was more likely to be comprehensive or explicitly stated in the text. Of note, there were hardly any observational studies aimed at evaluating benefits or effectiveness of polypharmacy. In addition, observational studies evaluating negative, but not directly harmful effects of polypharmacy such as cost, pill burden, and adherence were rare. These outcomes are better addressed by experimental studies.

### Strengths and limitations

The scoping review methodology supports a comprehensive scan of the literature and ensures a systematic approach. The comprehensive search of eight bibliographic databases from inception to current enabled us to find most of the published definitions and descriptions of pediatric polypharmacy. Our review spanned disease conditions, clinical settings, geographical location, and calendar time. Extracting definitions and descriptions of polypharmacy from both the text and numeric parts of the manuscripts provided both deliberate and indirect definitions of pediatric polypharmacy. A combination of quantitative and qualitative synthesis of the information we extracted enabled us to establish relationships between the text definitions and study characteristics, disease conditions, and medication categories.

Our study is not without limitations. Excluding non-English studies may not only have affected the geographical distribution of included studies, but it may have led to exclusion of definitions of pediatric polypharmacy. A closer look at the hand-searched studies revealed polypharmacy terms that were slightly different from those in our search strategy, which could have led to missing studies. For example, “combination treatment” instead of “combination pharmacotherapy” or “multiple classes” instead of “multiple medications”.

## Conclusions

More than 80% of the studies we reviewed defined polypharmacy as at least two medications with or without specifying duration. The most frequent and comprehensive definition of pediatric polypharmacy was the use of two or more concurrent medications or therapeutic classes for ≥1 days. Use of two or more concurrent medications for ≥31 days; use of two or more medications during a period of one year; and use of two or more medications during hospital stay were the other comprehensive definitions. Medication number and duration thresholds that define pediatric polypharmacy depend on the research question and context. Therefore, one uniform definition for pediatric polypharmacy may not be feasible because of the heterogeneity noted in the discussion. We provide guidance for defining pediatric polypharmacy which includes the following aspects: 1) number of medications or classes 2) whether they are concurrent or sequential, and 3) their duration.

We propose an epidemiological definition of pediatric outpatient polypharmacy as “the prescription or consumption of two or more distinct medications for at least one day”. This definition may be tailored and modified by further specifying the clinical setting, number, overlap duration, or classes of medications. The definition pediatric polypharmacy in inpatient settings needs further characterization. Future longitudinal studies should test the proposed definition of pediatric polypharmacy and characterize polypharmacy among hospitalized children. Systematic reviews and meta-analyses should be designed to examine specific aspects of pediatric polypharmacy such as inpatient setting, neonatal, or experimental pediatric polypharmacy. Pediatric polypharmacy researchers should build consensus around terminology, in the interim, the term polypharmacy should be used among key words and definitions.

## Supporting information

S1 Checklist PRISMA 2009 checklist. (DOC)

S1 File Study protocol. (DOCX)

S2 File Different database search strategies. (DOCX)

S1 Table Relationships between research questions and definitions of polypharmacy. Research questions:
Adverse events = drug-drug interactions, adverse reaction, adverse eventsMedication use = non-polypharmacy medication use questionsPrevalence = prevalence of polypharmacyOutcome of care = complications, death, prognostic measure, patient related outcomesDrug monitoring = drug levels, pharmacokinetics, pharmacodynamics.Threshold number of days were collapsed as follows:
≥1day category includes ≥1 day (51 studies) and ≥14 days (5)≥61 category includes ≥61 (5), ≥90 (3), ≥ 180 (4) and ≥365 (2)Sequential includes ≥1 year (6), 1 year (18), 2 years (7), and hospital stay (12).
(DOCX)

S2 Table All full text definitions of pediatric polypharmacy. AED = Antiepileptic drug
AP = Antipsychotic
LAS = Long-acting stimulants
SSRI = Selective serotonin reuptake inhibitor
CPT = Combined pharmacotherapy
ADHD = Attention-deficit/hyperactivity disorder
DP = Dispensed prescription
WHO = World Health Organization
VPA = Valproic acid
CBZ = Carbamazepine
PHT = Phenytoin
CZP = Clonazepam
SGA = Second generation antipsychotic
ART = Artesunate
AQ = Amodiaquine
LCM = Lacosamide
PDDI = Potential drug–drug interactions.
(DOCX)
