# Supplementary material for: Defining pediatric polypharmacy: A scoping review
Source: PLoS One. 2018 Nov 29;13(11):e0208047. doi: 10.1371/journal.pone.0208047 (PMC6264483; doi:10.1371/journal.pone.0208047)
Supplement: S1 File — (DOCX) [file pone.0208047.s002.docx]

**S1 File. Study Protocol**

**Definition of Pediatric Polypharmacy: A Scoping Review**

**SPECIFIC AIMS**

**Introduction**

Polypharmacy, the practice of administering or taking multiple medications for the treatment of one or several medical conditions, is a common problem among the elderly. However, with increasing complex disease conditions among pediatric patients, the prevalence of polypharmacy is also rising within this population of patients (Horace & Ahmed, 2015). Polypharmacy may lead to adverse drug effects, drug-to-drug interaction, hospitalization, poor medication adherence, mortality, resource wastage, burden of medical care, and high cost of healthcare. Polypharmacy is commonly defined as the use of 5 or more prescribed medications in adults and 2 or more medications in children. But the definition of polypharmacy varies depending on disease, medication class, duration of medication administration, or healthcare setting. There is a need to examine the differences among these interpretations of pediatric polypharmacy in order to recommend cohesive and comprehensive definitions that will lead to comparable measures across studies for specific pediatric populations. Consistent definition of polypharmacy will lead to consistent assessment of its clinical importance when studying associated outcomes.

Conducting a scoping review of the definition(s) of pediatric polypharmacy is therefore, warranted. A scoping review is a form of knowledge synthesis that addresses an exploratory research question aimed at mapping key concepts, types of evidence, and gaps in research related to a defined area or field by systematically searching, selecting, and synthesizing existing knowledge (Colquhoun et al., 2014). With the goal of examining the extent, range, and nature of pediatric polypharmacy, we seek to establish how polypharmacy has been defined, and to adopt or propose a definition or sub population-specific definitions for subsequent use.

**Research Question**

We set out with a broad research question expecting that the definition of polypharmacy in children will vary according to disease, health delivery setting, study aims, or type of medication.

What are the definitions of pediatric polypharmacy?

Specifically we aim to:

1. summarize the scope of research on pediatric polypharmacy among children from birth to 18 years of age
2. describe the definition(s) of pediatric polypharmacy which have been used in different conditions and across settings over the period covered by the databases of indexed citations
3. determine definition-specific prevalence of pediatric polypharmacy
4. adopt or propose subpopulation-specific definitions of pediatric polypharmacy

**BACKGROUND**

An inevitable consequence of providing adequate evidence-based medical care for patients with multiple chronic conditions (MCC) is polypharmacy, commonly defined as use of 5 or more prescribed medications in adults and 2 or more medications in children (Feinstein et al., 2015). Other definitions of polypharmacy consider duration, continuity, and accumulation of medications (Fincke et al., 2005; Chen et al., 2011). Apart from psychotropic polypharmacy (Constantine et al., 2010; Spencer et al., 2013) and inpatient polypharmacy (Feudtner et al., 2012, Krähenbühl-Melcher et al., 2007), pediatric outpatient polypharmacy has hardly been studied (Chai et al., 2012; Feinstein et al., 2015). Feinstein and colleagues’ recent study among Colorado Medicaid beneficiaries revealed that 35% of children were exposed to >=2 concurrent medications for at least 1 day. Compared to the rest of the children, those with MCC were exposed to more medications over longer periods including those at heightened risk of causing harm such as psychotropic agents, anticonvulsants, cardiovascular agents and opioids. Risks of polypharmacy include adverse drug effects, drug-to-drug interaction, hospitalization, poor adherence to medications, death, resource wastage, burden of medical care, and high cost of healthcare (Guthrie et. al, 2011). There is a need to conduct a scoping review of the available literature for the definitions of pediatric polypharmacy in an effort to unify the definition(s) for comparable measurement across studies which may differ by subsets of pediatric populations.

A scoping review is a form of knowledge synthesis that addresses an exploratory research question aimed at mapping key concepts, types of evidence, and gaps in research related to a defined area or field by systematically searching, selecting, and synthesizing existing knowledge (Colquhoun et al., 2014). This definition is a modification of an earlier definition of scoping review proposed by Arksey and O’Malley (Arksey and O’Malley, 2005) and enhanced by Daudt and colleagues (Daudt et al., 2013). Arksey and O’Malley identified four reasons for conducting a scoping study: 1) to examine the extent, range and nature of research activity, 2) to determine the value of undertaking a full systematic review, 3) to summarize and disseminate research findings, and 4) to identify research gaps in the existing literature (Arksey and O’Malley, 2005). They further proposed a six step framework of conducting a scoping review including: 1) identifying the research question, 2) identifying relevant studies, 3) study selection, 4) charting the data, 5) collating, summarizing, and reporting the results, and, an optional phase, 6) consultation (Arksey and O’Malley, 2005). This framework has since been enhanced, clarified, or operationalized by other researchers (Levac et al., 2010; Daudt et al., 2013; Colquhoun et al., 2014; Pham et al., 2014; Pham, 2014).

Like a systematic review, a scoping review uses transparent and reproducible processes to define a research question, search for studies, and synthesize findings. However, compared to a systematic review, a scoping review tends to address a broader research question, may develop inclusion/exclusion criteria a posteriori or in an iterative fashion as the group reviews the existing literature, may not assess quality of studies, and literature synthesis is more qualitative or descriptive than quantitative (Armstrong et al., 2011).

The purpose of the proposed study is to examine the extent, range, and nature of pediatric polypharmacy. We further seek to establish how pediatric polypharmacy has been defined in different settings, and to adopt or propose definitions of polypharmacy for subsequent use. Our study is based on reasons 1 and 3 among those proposed by Arksey and O’Malle for conducting scoping reviews (Arksey and O’Malley, 2005).

**RATIONALE AND SIGNIFICANCE**

A scoping review is the best method for establishing the definitions of pediatric polypharmacy since it uses a reproducible methodological approach similar to a systematic review to identify all studies on a topic and reduce the chance of publication bias as much as possible in selecting studies. But it allows for an iterative process of selecting studies and is more flexible on the quality of studies included to enhance the descriptive understanding of the literature on a topic.This scoping review will establish the available literature on pediatric polypharmacy and the definitions of pediatric polypharmacy under different circumstances for use in subsequent inquiries in pediatric polypharmacy. These inquiries will include characterizing polypharmacy in Medicaid claims data, assessing its impact on outcomes such as service utilization and cost of care, and its management among providers, patients, and caregivers.

**METHODS**

**Study Design**

We shall conduct a scoping review of the definition(s) of pediatric polypharmacy according to the framework proposed by Arksey and O’Malley (Arksey and O’Malley, 2005), and enhanced by Levac and colleagues (Levac et al., 2010; Colquhoun et al., 2014). The framework involves six phases: 1) identifying the research question, 2) identifying relevant studies, 3) study selection, 4) charting the data, 5) collating, summarizing, and reporting the results, and, an optional phase, 6) consultation. The procedures of conducting a scoping review described here are adapted from Pham and colleagues’ manuscript and dissertation about scoping review (Pham t al., 2014, Pham 2014).

**Data Sources**

We shall search PubMed (biomedical science), Ovid Medline, Excerpta Medica dataBASE (EMBASE) (biomedical and pharmacological), CINAHL (nursing and allied health)**,** PsycINFO (psychology), Web of Science Core Collection (multidisciplinary)**,** and ProQuest Dissertations and Theses A&I for the studies to be reviewed. We shall then hand search bibliographies of studies included in the review for articles that may not be identified by our search strategy.

**Identification of Relevant Studies**

The search will include controlled vocabulary and free text terms for the following topics: (pediatrics or children or youth or adolescents) AND (polypharmacy or multiple medications). Childhood will be defined as birth to 18 years of age. All studies selected through the initial search will be included in the review if they are primary studies about polypharmacy in children. Screening of potential studies will be conducted using title, abstract, and text review forms (appendix 1) approved by our research team. Both the title, abstract, and text review forms will be pilot tested with 20 titles/abstracts and 10 full text articles and edited accordingly.

**Study Selection**

All titles and abstracts identified through the initial search will be screened by two independent reviewers for inclusion using a screening form. If any one person includes a study at title review, it will move on to abstract review. Studies not written about polypharmacy in children <18 years of age or not written in English will be excluded. At abstract and full text review, both reviewers must reach consensus on whether to include the study or not. Disagreement in study selection will be resolved by consensus through a telephone, skype, or in-person meeting held after blocks of 50 abstracts or 20 full text reviews. Since this is a scoping review and we may have many articles included for data abstraction, we will use a random sample of the included studies and review articles on this topic to search bibliographies as part of a hand search to ensure no major studies are missing. A simple random sample of 20 and 5 original and review articles, respectively, will be performed with all selected articles in the sampling frame. If we end up with <50 included studies, we will consider reviewing all bibliographies of included studies as part of our hand search. Studies found through this process will also be screened for possible inclusion. Depending on time interval between the initial search and publishing the results, the search may be updated. Search terms and the text review form may be revised during study implementation if need arises. Quality of selected studies will not be appraised since this is a descriptive review of the definition of polypharmacy as opposed to comparing interventions or evaluating intervention outcomes.

**Charting the Data**

Full text of each study passing the screening phase will be reviewed for data extraction by the first reviewer. A second reviewer will then read the study and check the data extraction form (appendix 2) for accuracy. Any errors or discrepancies at this stage will be corrected and the form saved for analysis. The information to be extracted from the studies and charted on the form will include: first author, year of publication, country where the study was conducted, age-group studied, aim of study, type of study, whether polypharmacy was defined (explicitly or implicitly), definition of polypharmacy, disease(s) studied, medications studied (individual/class), context (inpatient/outpatient).

**Collating, Summarizing, and Reporting Results**

The data will be coded and the definitions of polypharmacy grouped according to type, such as disease(s) or medication classes. Counts and proportions will be used to summarize categorical data. Descriptive summaries will be presented as tables and figures. If we identify any relevant material for qualitative (as opposed to descriptive) analysis during preliminary data abstraction, we will then create coding templates to identify relevant themes. We will further modify our methods to describe a more in depth and appropriate qualitative thematic analysis should this issue arise. Overall and/or contextual definitions of polypharmacy will be summarized, and definitions of polypharmacy pertinent to subpopulations context will be selected or adapted. Prevalence of pediatric polypharmacy will be described and, if deemed necessary, analyzed to provide overall estimate(s). Internal validation of the abstract and study selection between reviewers will be summarized using the kappa statistic.

EndNote software will be used for citation management including searching databases for potential studies, importing references from the databases, deduplication of references, organizing and tagging the references and abstracts. The references, abstracts, and full text articles will be imported into EPPI-Reviewer 4 software (Thomas Brunton, 2009) for screening, data extraction, collating, summarizing, and reporting. Numerical analysis will be conducted using SAS version 9.4.

**Consultation**

Consultation with some authors of articles cited in this proposal, pediatricians, family practitioners, nurses, and pharmacists will be conducted during the planning, implementation, and report writing phases (appendix 3). The team of 10-15 stakeholders will be constituted through a snowball approach by our team members contacting individuals they know as experts or have interest in pediatric polypharmacy. Those people, and some authors of manuscripts cited in this proposal will be requested to contact other experts until we reach a maximum of 15 people to consult. Input will be sought about the research question, search terms, inclusion/exclusion criteria, databases, preliminary results, and final report. The input will be written comments in response to a question related to each of the areas and stages of consultation. The research team will synthesize these comments and incorporate them in the different stages of the research process. Stakeholders may be co-authors of the manuscript if more deeply involved beyond consultation, otherwise they will be acknowledged for their contribution. They will also receive a summary of the final report.

**QUALITY ASURANCE AND QUALITY CONTROL**

We shall build in quality assurance and quality control measures to ensure both internal and external validity of this study. Our detailed protocol specifying the research question, databases to be searched, search terms, two independent reviewers, reproducible methodology, and inclusion of stakeholder input is the first line of quality assurance. Training of reviewers, pilot testing instruments, regular meetings, and internal validity analysis (kappa statistic) are some of the additional quality control measures to be used.

**THE RESEARCH TEAM**

We are a multidisciplinary team of pediatricians, internists, pharmacists, epidemiologists, social scientists, mental health specialists, nurses, librarians, and health services researchers in the field of chronic childhood diseases and their management. Our team is constituted of investigators who will implement the study and stakeholders who will serve as study consultants.

**Investigators**

Our team will be led by **Dr. Paul Bakaki**, an epidemiologist and health services researcher with expertise in large data based outcomes research, research synthesis, and field research in chronic disease of both children and adults. This is the first of a series of studies he will lead to consolidate his research portfolio in chronic childhood disease management including management of chemotherapeutics through a career development grant. He will lead and participate in all research activities.

**Dr.** **Lawrence Kleinman** is a pediatrician, Director of the Center for Child Health and Policy, and Vice Chair, Child Health and Policy at Rainbow Babies & Children's Hospital and Case Western Reserve University School of Medicine. He is an expert health services and child healthcare quality measurement research. He has conducted scoping reviews in the area of pediatric quality measures. As the primary research mentor of Dr. Paul Bakaki, he will oversee the implementation of this research project. He will provide guidance about scoping review to Dr. Bakaki and the rest of the research team.

**Dr. Shari Bolen** is an internist and associate professor of medicine, epidemiology, and biostatistics at MetroHealth Medical Center and Case Western Reserve University, Cleveland, Ohio. Her expertise is teaching and conducting systematic reviews and meta-analyses as well as comparative effectiveness research. She will be involved in the whole project cycle from protocol development to dissemination. Additionally she will guide Dr. Bakaki through the intricate steps of designing, conducting, analyzing, and publishing a scoping review through tutoring, assigning readings, and critiquing his work.

**Dr. Negar Golchin** is a practicing pharmacist and clinical instructor of pharmacy and public health students at the University of Washington, Seattle, WA. She has expertise in improving drug adherence, polypharmacy management, spontaneous adverse drug reaction reporting, and pharmacoepidemiology studies. She will be involved in the whole research cycle including protocol development and reviewing articles.

**Dr. Alexis Horace**is an assistant professor at the University of Louisiana at Monroe, College of Health & Pharmaceutical Sciences, School of Pharmacy. She has extensive experience in ambulatory care specializing in HIV/AIDS and also has a special interest in pediatrics. She has conducted a systematic review on pediatric polypharmacy from the pharmacists’ perspective. She will serve both as a stakeholder and reviewer.

**Jennifer Staley**, MLIS, is a medical librarian at Rainbow Babies and Children’s Hospital, Cleveland, Ohio and a doctoral student concentrating in Health Informatics at Kent State University, Kent, OH. She will create and translate the search queries into the different databases and manage the search results.

**To be named** is a graduate student research assistant who, in pairs, with Paul Bakaki, Negar Golchin, or Alexis Horace will review qualifying studies and complete screening and data extraction forms.

**List of Stakeholders**

| **Name** | **Profession** | **Institution** | **Expertize** |
| --- | --- | --- | --- |
| Dr. Mai Pham | Public Health | University of Guelph, Guelph, Ontario | Scoping Review |
| Dr. Jamie Feinstein | Pediatrics | University of Colorado School of Medicine, Aurora, CO | Complex Chronic Conditions, Polypharmacy |
| Dr. Cynthia Fontanella | Mental Health, Social Science | Ohio State University, Columbus, OH | Psychopharmacology |
| Dr. Alexis Horace | Pharmacy | University of Louisiana, Monroe College of Pharmacy, Baton Rouge, LA | Ambulatory Care |
| Dr. Elia Pestana-Knight | Pediatric Neurology Epilepsy | Cleveland Clinic, Cleveland, OH | Medical Home, Outcomes Research |
| Dr. Sharon Meropol | Pediatrics, Pharmacoepidemiology | University Hospitals, Cleveland, OH | Antibiotic Resistance |
| Prof. Almut Winterstein | Pharmacoepidemiology | University of Florida, College of Pharmacy, Gainesville, FL | Drug Safety and Effectiveness |
| Dr. Joseph Calabrese | Psychiatry | Case Western Reserve University, Cleveland, OH | Bipolar Disorders, Medication Development |
| Prof. Faye Gary | Nursing, Childhood Mental Health | Case Western Reserve University, School of Nursing, Cleveland, OH | Mental Disorders among Vulnerable Children |

**CAREER DEVELOPMENT**

This is the first study to be conducted by Dr. Paul Bakaki, his collaborators, and mentors towards developing a research career that will lead to improvement of the health of children with chronic disease. This study describes the scope of research and definitions of pediatric polypharmacy, an inevitable consequence of chronic disease. The definition(s) of pediatric outpatient polypharmacy identified in this study will be used to characterize polypharmacy in Medicaid claims data so as to assess its impact on service utilization and cost of care. The scope of research in pediatric polypharmacy will inform future research by our team and other researchers.

Through the implementation of this study, Dr. Bakaki will master several skills including, but not limited to, scoping review research methodology and research team management.

**STUDY TIMELINE**

| **Activity** | **Year 1 (2016-2017)** | | | | | | | | | | |
| --- | --- | --- | --- | --- | --- | --- | --- | --- | --- | --- | --- |
|  | Jul | Aug | Sep | Oct | Nov | Dec | Jan | Feb | Mar | Apr | May |
| Proposal development & submission | X | X | X |  |  |  |  |  |  |  |  |
| Constitute research team & stakeholders |  | X | X |  |  |  |  |  |  |  |  |
| Hire research assistant |  |  | X |  |  |  |  |  |  |  |  |
| Constitute review team |  | X | X |  |  |  |  |  |  |  |  |
| Training of review team |  |  | X |  |  |  |  |  |  |  |  |
| Pilot search terms, screening, & extraction forms |  |  | X |  |  |  |  |  |  |  |  |
| Search for relevant studies |  |  |  | X | X |  |  |  |  |  |  |
| Compilation of citations and removal of duplicates |  |  |  | X | X |  |  |  |  |  |  |
| Title and abstract screening review |  |  |  | X | X |  |  |  |  |  |  |
| Procure full text articles |  |  |  | X | X |  |  |  |  |  |  |
| Text review and confirmation of relevance |  |  |  |  | X | X |  |  |  |  |  |
| Data charting and analysis |  |  |  |  |  | X | X | X |  |  |  |
| Collate, summarize data, and write report |  |  |  |  |  |  |  | X | X | X |  |
| Consult stakeholders |  | X | X |  |  |  |  | X |  | X |  |
| Team meetings |  |  | X | X | X | X | X | X | X | X | X |
| Dissemination (manuscript submission) |  |  |  |  |  |  |  |  |  |  | X |
| Dissemination (conference) |  |  |  |  |  |  |  |  |  |  | X |

**LITERATURE CITED**

Armstrong R., Hall B. J., Doyle J., & Waters E. (2011). ‘Scoping the scope’of a cochrane review. *Journal of Public Health*, *33*(1), 147-150.

Arksey H., & O'Malley L. (2005). Scoping studies: towards a methodological framework. *International journal of social research methodology*, *8*(1), 19-32.

Chen H., Patel A., Sherer J., & Aparasu R. (2011). The definition and prevalence of pediatric psychotropic polypharmacy. *Psychiatric services*.

Chai G., Governale L., McMahon A. W., Trinidad J. P., Staffa J., & Murphy D. (2012). Trends of outpatient prescription drug utilization in US children, 2002–2010. *Pediatrics*, *130*(1), 23-31.

Colquhoun H. L., Levac D., O'Brien K. K., Straus S., Tricco A. C., Perrier L., ... & Moher D. (2014). Scoping reviews: time for clarity in definition, methods, and reporting. *Journal of clinical epidemiology*, *67*(12), 1291-1294.

Constantine R. J., Boaz T., & Tandon R. (2010). Antipsychotic polypharmacy in the treatment of children and adolescents in the fee-for-service component of a large state Medicaid program. *Clinical therapeutics*,*32*(5), 949-959.

Daudt H. M., Van Mossel C., & Scott S. J. (2013). Enhancing the scoping study methodology: a large, inter-professional team’s experience with Arksey and O’Malley’s framework. *BMC medical research methodology*, *13*(1), 1.

Feinstein J. A., Feudtner C., Valuck R. J., & Kempe A. (2015). The depth, duration, and degree of outpatient pediatric polypharmacy in Colorado fee‐for‐service Medicaid patients. *Pharmacoepidemiology and Drug Safety*, *24*(10), 1049-1057.

Feudtner C., Dai D., Hexem K. R., Luan X., & Metjian T. A. (2012). Prevalence of polypharmacy exposure among hospitalized children in the United States. *Archives of pediatrics & adolescent medicine*, *166*(1), 9-16.

Fincke B. G., Snyder K., Cantillon C., Gaehde S., Standring P., Fiore L., ... & Gagnon D. R. (2005). Three complementary definitions of polypharmacy: methods, application and comparison of findings in a large prescription database. *Pharmacoepidemiology and drug safety*, *14*(2), 121-128.

Fontanella C. A., Warner L. A., Phillips G. S., Bridge J. A., & Campo J. V. (2014). Trends in psychotropic polypharmacy among youths enrolled in Ohio Medicaid, 2002–2008. *Psychiatric Services*.

Gallacher K. I., Batty G. D., McLean G., Mercer S. W., Guthrie B., May C. R., ... & Mair F. S. (2014). Stroke, multimorbidity and polypharmacy in a nationally representative sample of 1,424,378 patients in Scotland: implications for treatment burden. *BMC medicine*, *12*(1), 1.

Guthrie B., McCowan C., Davey P., Simpson C. R., Dreischulte T., & Barnett K. (2011). High risk prescribing in primary care patients particularly vulnerable to adverse drug events: cross sectional population database analysis in Scottish general practice. *Bmj*, *342*, d3514.

Horace A. E., & Ahmed F. (2015). Polypharmacy in pediatric patients and opportunities for pharmacists' involvement. *Integrated Pharmacy Research and Practice*, *4*, 113-126.

Levac D., Colquhoun H., & O'Brien K. K. (2010). Scoping studies: advancing the methodology. *Implementation Science*, *5*(1), 1.

Pham M. T., Rajić A., Greig J. D., Sargeant J. M., Papadopoulos A., & McEwen S. A. (2014). A scoping review of scoping reviews: advancing the approach and enhancing the consistency. *Research synthesis methods*,*5*(4), 371-385.

Pham M. (2014). *Advancing the Use of Knowledge Synthesis to Inform Policy and Decision Making in Agri-food Public Health* (Doctoral dissertation, John Wiley & Sons, Ltd.).

Spencer D., Marshall J., Post, B., Kulakodlu M., Newschaffer C., Dennen T., ... & Jain A. (2013). Psychotropic medication use and polypharmacy in children with autism spectrum disorders. *Pediatrics*, *132*(5), 833-840.

Thomas J, Brunton J. (2009). EPPI-Reviewer 3.0: Analysis and Management of Data for Research Synthesis. London: EPPICentre Software, Social Science Research Unit, Institute of Education, 2009.

**APPENDICES**

**Appendix 1: Study Screening Form**

*Please complete the following sections to determine if this study should be included.*

**Appendix 1a: Title Review Form**

Does this article potentially apply to pediatric polypharmacy?

- Yes or possibly
- No (stop here)

**Appendix 1b: Abstract Review Form**

Please exclude the study if it (check all that apply):

- Does not apply to any of the questions asked in this scoping review (for instance, pediatric polypharmacy not addressed)
- Only includes adults (>=18 years old) or data not stratified by pediatric and adult populations
- Not a primary study
- Not written in English
- Other (specify) _______________________________________________

If did not meet any exclusion criteria, then click below:

- Include study for full article review – choose this if unclear based on the abstract

**Appendix 1c: Full Article Review Form**

Please exclude the study if it (check all that apply):

- Does not apply to any of the questions asked in this scoping review (for instance, pediatric polypharmacy not addressed)
- Only includes adults (>=18 years old) or data not stratified by pediatric and adult populations
- Not a primary study
- Not written in English
- Other (specify) _______________________________________________

If did not meet any exclusion criteria, then click below:

- Include study for full article review

**Appendix 2: Data Extraction Form**

1. *Before completing this form for the first time, please read through it several times to familiarize yourself with the sections, questions, and their sequence.*
2. *Whenever you are completing the form for a specific study/report, please skim the whole article before completing the first section (Study Type)*.

| 1. **Study Type** | | |
| --- | --- | --- |
| **Question** | **Response** | **Directions** |
| 1. Publication type | 1. Journal article 2. Conference proceeding 3. Dissertation 4. Institutional report 5. Other (specify) ----------- | Please select one. |
| 1. If journal article, please, check originality | 1. Original 2. Review 3. Editorial 4. Other (specify) ------------- |  |
| 1. **Text Review** | | |
| 1. What was the purpose of the study? | ------------------------ | Please copy and paste the purpose (or aim) of the study into the text box |
| 1. Which country was the study conducted? | 1. ------------------------- 2. ------------------------- 3. ------------------------- 4. Country not named | Write name(s) of country(s) in text box or check option b. |
| 1. What age group was studied? | ------------------------- | Write age group (range) studied |
| 1. Was polypharmacy defined? | 1. Yes, explicitly 2. Yes implicitly 3. No | Check a. if polypharmacy (or other terms) was directly defined. Check b if definition is indirect. |
| 1. What terms were used to describe polypharmacy? | 1. Polypharmacy 2. Multiple Medications 3. Multiple Drugs 4. Concomitant Medications 5. Co-Medications 6. Combined Pharmacotherapy 7. Other (Specify) ------------- | You may check more than one. |
| 1. What was the definition of polypharmacy? | 1. --------------- 2. --------------- 3. --------------- 4. Not defined | Copy and paste definition of polypharmacy in text box. Check d if it was not defined. Include all definitions if more than 1. |
| 1. What was the source of the definition(s) of polypharmacy? | 1. -------------- 2. Original | Write/copy & paste source e.g. a citation. Check b if it was authors’ original definition |
| 1. What was the prevalence of polypharmacy? | 1. Overall --------------------- 2. Specific 1------------------   Category 1-----------------   1. Specific 2------------------   Category 2 ---------------- |  |
| 1. Please name disease(s) studied | 1. ------------- 2. Not mentioned | State names of specific diseases if stated in the article |
| 1. What group of diseases were studied? | 1. Mental health 2. Somatic diseases 3. Both mental and somatic diseases 4. Diseases not mentioned | Mental conditions include: |
| 1. What groups (classes) of medications were studied? | 1. Psychotropic medications 2. Somatic medications 3. Both psychotropic and somatic medications 4. Not mentioned | Other terms for psychotropic medications are: |
| 1. Please name classes or individual medications studied | 1. ------------ 2. Not mentioned | State names (classes) of specific medications studied |
| 1. What was the study setting? | 1. Outpatient (OP) 2. Inpatient (IP) 3. Both OP & IP 4. Other (specify) -------- 5. Setting not mentioned |  |
| 1. What was the main finding in relation to the aim in question 3? | 1. -------------- 2. Not mentioned | Please copy and paste main finding |
| 1. Want were the other findings? | 1. --------------- 2. Not mentioned | Please copy and paste other findings |
| 1. Please provide any other comments | --------- |  |
| 1. Please write your initials | ---------- |  |

**Definition of Pediatric Polypharmacy: A Scoping Review**

**Appendix 3: Stakeholder Request Form**

*Please read, complete, and submit this form to the research office.*

We are a team of researchers in the field of childhood chronic disease and medication use conducting a scoping study of pediatric polypharmacy to map the extent of research in the this field and to describe the definition(s) of pediatric polypharmacy used by researchers. Polypharmacy is the practice of administering or taking multiple medications for the treatment of one or several medical conditions. Our team is led by Dr. Lawrence Kleinman and Dr. Paul Bakaki of Case Western Reserve University, Cleveland, OH. The team has three non-mutually exclusive categories of members:

1. **Reviewers**: Pair up to conduct title, abstract, and full text review of at least 15 articles following a detailed protocol.
2. **Investigators**: Develop, amend, and oversee the implementation of the study protocol. They write, critically review, and approve the publication of manuscripts resulting from our research activities.
3. **Stakeholders**: These are content experts in childhood chronic disease or chemotherapeutics, or they are providers, policymakers, program managers. They provide feedback to the research team about specific issues at different stages of the research cycle.

Individuals in any of the above categories may co-author manuscripts arising from our research if they fulfill requirements for authorship**.** Otherwise they are acknowledged for their input and receive a summary of the final report.

We invite you to be a stakeholder on a scoping study mapping the extent of research and definition of polypharmacy.

As a stakeholder on this study you are required to provide written comments on the following issues at the specified stages:

**Protocol development**

- Research question
- Databases to be searched
- Search terms to be used

**Preliminary results**

- Comments & suggestions regarding preliminary findings in relation to research question.

**Final results**

- Comments & suggestions regarding final findings in relation to research question.

Are you willing to be a stakeholder in the study “Definition of Pediatric Polypharmacy: A Scoping Review”?

1. Yes
2. No

If Yes please provide your

Names _________________________________________________________

Email(s) _______________________ / ________________________________

Telephone(s) ______________________ / _____________________________
