# Supplementary material for: Defining pediatric polypharmacy: A scoping review
Source: PLoS One. 2018 Nov 29;13(11):e0208047. doi: 10.1371/journal.pone.0208047 (PMC6264483; doi:10.1371/journal.pone.0208047)
Supplement: S2 File — (DOCX) [file pone.0208047.s003.DOCX]

**S2 File.** **Different Database Search Strategies**

**Searches run on 07/11/2017**

**Ovid MEDLINE**(R) Epub Ahead of Print, In-Process & Other Non-Indexed Citations, Ovid MEDLINE(R) Daily and Ovid MEDLINE(R) <1946 to Present> Search Strategy:

--------------------------------------------------------------------------------

1 exp Polypharmacy/ (3589)

2 ((prescribe* or prescription*) adj10 multiple drug*).ti,ab,kf. (89)

3 (hyperpolypharmacy or hyper-polypharmacy or hyper polypharmacy or polypharmacy or poly-pharmacy or poly pharmacy or polytherapy or poly-therapy or poly therapy or poly-medication or polymedication or poly medication or multiple medication* or multiple prescription* or combination pharmacotherapy).ti,ab,kf. (8521)

4 1 or 2 or 3 (10366)

5 limit 4 to "all child (0 to 18 years)" (1515)

6 exp Child, Hospitalized/ (6276)

7 4 and 6 (3)

8 (child* or infant* or neonate* or toddler* or adolescent* or teen* or pediatric* or paediatric* or school or boy* or girl* or baby or babies or newborn* or juvenile* or minors).ti,ab,kf. (2141983)

9 4 and 8 (1010)

10 5 or 7 or 9 (1833)

11 remove duplicates from 10 (1757)

**PubMed**

| [#10](https://www.ncbi.nlm.nih.gov/pubmed/advanced) | [Add](https://www.ncbi.nlm.nih.gov/pubmed/advanced) | Search ((((((Polypharmacy[MeSH Terms]) OR ((hyperpolypharmacy[Text Word] OR hyper-polypharmacy[Text Word] OR hyper polypharmacy[Text Word] OR polypharmacy[Text Word] OR poly-pharmacy[Text Word] OR poly pharmacy[Text Word] OR polytherapy[Text Word] OR poly-therapy[Text Word] OR poly therapy[Text Word] OR poly-medication[Text Word] OR polymedication[Text Word] OR poly medication[Text Word] OR multiple medication*[Text Word] OR multiple prescription*[Text Word] OR combination pharmacotherapy[Text Word])))) AND (((Infant[MeSH Terms]) OR Child[MeSH Terms]) OR Adolescent[MeSH Terms]))) OR ((((Polypharmacy[MeSH Terms]) OR ((hyperpolypharmacy[Text Word] OR hyper-polypharmacy[Text Word] OR hyper polypharmacy[Text Word] OR polypharmacy[Text Word] OR poly-pharmacy[Text Word] OR poly pharmacy[Text Word] OR polytherapy[Text Word] OR poly-therapy[Text Word] OR poly therapy[Text Word] OR poly-medication[Text Word] OR polymedication[Text Word] OR poly medication[Text Word] OR multiple medication*[Text Word] OR multiple prescription*[Text Word] OR combination pharmacotherapy[Text Word])))) AND Child, Hospitalized[MeSH Terms])) OR ((((Polypharmacy[MeSH Terms]) OR ((hyperpolypharmacy[Text Word] OR hyper-polypharmacy[Text Word] OR hyper polypharmacy[Text Word] OR polypharmacy[Text Word] OR poly-pharmacy[Text Word] OR poly pharmacy[Text Word] OR polytherapy[Text Word] OR poly-therapy[Text Word] OR poly therapy[Text Word] OR poly-medication[Text Word] OR polymedication[Text Word] OR poly medication[Text Word] OR multiple medication*[Text Word] OR multiple prescription*[Text Word] OR combination pharmacotherapy[Text Word])))) AND ((child*[Text Word] OR infant*[Text Word] OR neonate*[Text Word] OR toddler*[Text Word] OR adolescent*[Text Word] OR teen*[Text Word] OR pediatric*[Text Word] OR paediatric*[Text Word] OR school[Text Word] OR boy*[Text Word] OR girl*[Text Word] OR baby[Text Word] OR babies[Text Word] OR newborn*[Text Word] OR juvenile*[Text Word] OR minors[Text Word]))) | [1748](https://www.ncbi.nlm.nih.gov/pubmed/?cmd=HistorySearch&querykey=10) | 12:36:41 |
| --- | --- | --- | --- | --- |
| [#9](https://www.ncbi.nlm.nih.gov/pubmed/advanced) | [Add](https://www.ncbi.nlm.nih.gov/pubmed/advanced) | Search (((Polypharmacy[MeSH Terms]) OR ((hyperpolypharmacy[Text Word] OR hyper-polypharmacy[Text Word] OR hyper polypharmacy[Text Word] OR polypharmacy[Text Word] OR poly-pharmacy[Text Word] OR poly pharmacy[Text Word] OR polytherapy[Text Word] OR poly-therapy[Text Word] OR poly therapy[Text Word] OR poly-medication[Text Word] OR polymedication[Text Word] OR poly medication[Text Word] OR multiple medication*[Text Word] OR multiple prescription*[Text Word] OR combination pharmacotherapy[Text Word])))) AND ((child*[Text Word] OR infant*[Text Word] OR neonate*[Text Word] OR toddler*[Text Word] OR adolescent*[Text Word] OR teen*[Text Word] OR pediatric*[Text Word] OR paediatric*[Text Word] OR school[Text Word] OR boy*[Text Word] OR girl*[Text Word] OR baby[Text Word] OR babies[Text Word] OR newborn*[Text Word] OR juvenile*[Text Word] OR minors[Text Word])) | [1748](https://www.ncbi.nlm.nih.gov/pubmed/?cmd=HistorySearch&querykey=9) | 12:33:03 |
| [#8](https://www.ncbi.nlm.nih.gov/pubmed/advanced) | [Add](https://www.ncbi.nlm.nih.gov/pubmed/advanced) | Search (child*[Text Word] OR infant*[Text Word] OR neonate*[Text Word] OR toddler*[Text Word] OR adolescent*[Text Word] OR teen*[Text Word] OR pediatric*[Text Word] OR paediatric*[Text Word] OR school[Text Word] OR boy*[Text Word] OR girl*[Text Word] OR baby[Text Word] OR babies[Text Word] OR newborn*[Text Word] OR juvenile*[Text Word] OR minors[Text Word]) | [3884961](https://www.ncbi.nlm.nih.gov/pubmed/?cmd=HistorySearch&querykey=8) | 12:32:46 |
| [#7](https://www.ncbi.nlm.nih.gov/pubmed/advanced) | [Add](https://www.ncbi.nlm.nih.gov/pubmed/advanced) | Search (((Polypharmacy[MeSH Terms]) OR ((hyperpolypharmacy[Text Word] OR hyper-polypharmacy[Text Word] OR hyper polypharmacy[Text Word] OR polypharmacy[Text Word] OR poly-pharmacy[Text Word] OR poly pharmacy[Text Word] OR polytherapy[Text Word] OR poly-therapy[Text Word] OR poly therapy[Text Word] OR poly-medication[Text Word] OR polymedication[Text Word] OR poly medication[Text Word] OR multiple medication*[Text Word] OR multiple prescription*[Text Word] OR combination pharmacotherapy[Text Word])))) AND Child, Hospitalized[MeSH Terms] | [3](https://www.ncbi.nlm.nih.gov/pubmed/?cmd=HistorySearch&querykey=7) | 12:32:33 |
| [#6](https://www.ncbi.nlm.nih.gov/pubmed/advanced) | [Add](https://www.ncbi.nlm.nih.gov/pubmed/advanced) | Search Child, Hospitalized[MeSH Terms] | [6127](https://www.ncbi.nlm.nih.gov/pubmed/?cmd=HistorySearch&querykey=6) | 12:32:18 |
| [#5](https://www.ncbi.nlm.nih.gov/pubmed/advanced) | [Add](https://www.ncbi.nlm.nih.gov/pubmed/advanced) | Search (((Polypharmacy[MeSH Terms]) OR ((hyperpolypharmacy[Text Word] OR hyper-polypharmacy[Text Word] OR hyper polypharmacy[Text Word] OR polypharmacy[Text Word] OR poly-pharmacy[Text Word] OR poly pharmacy[Text Word] OR polytherapy[Text Word] OR poly-therapy[Text Word] OR poly therapy[Text Word] OR poly-medication[Text Word] OR polymedication[Text Word] OR poly medication[Text Word] OR multiple medication*[Text Word] OR multiple prescription*[Text Word] OR combination pharmacotherapy[Text Word])))) AND (((Infant[MeSH Terms]) OR Child[MeSH Terms]) OR Adolescent[MeSH Terms]) | [1437](https://www.ncbi.nlm.nih.gov/pubmed/?cmd=HistorySearch&querykey=5) | 12:32:05 |
| [#4](https://www.ncbi.nlm.nih.gov/pubmed/advanced) | [Add](https://www.ncbi.nlm.nih.gov/pubmed/advanced) | Search ((Infant[MeSH Terms]) OR Child[MeSH Terms]) OR Adolescent[MeSH Terms] | [3181766](https://www.ncbi.nlm.nih.gov/pubmed/?cmd=HistorySearch&querykey=4) | 12:31:57 |
| [#3](https://www.ncbi.nlm.nih.gov/pubmed/advanced) | [Add](https://www.ncbi.nlm.nih.gov/pubmed/advanced) | Search (Polypharmacy[MeSH Terms]) OR ((hyperpolypharmacy[Text Word] OR hyper-polypharmacy[Text Word] OR hyper polypharmacy[Text Word] OR polypharmacy[Text Word] OR poly-pharmacy[Text Word] OR poly pharmacy[Text Word] OR polytherapy[Text Word] OR poly-therapy[Text Word] OR poly therapy[Text Word] OR poly-medication[Text Word] OR polymedication[Text Word] OR poly medication[Text Word] OR multiple medication*[Text Word] OR multiple prescription*[Text Word] OR combination pharmacotherapy[Text Word])) | [9852](https://www.ncbi.nlm.nih.gov/pubmed/?cmd=HistorySearch&querykey=3) | 12:31:25 |
| [#2](https://www.ncbi.nlm.nih.gov/pubmed/advanced) | [Add](https://www.ncbi.nlm.nih.gov/pubmed/advanced) | Search (hyperpolypharmacy[Text Word] OR hyper-polypharmacy[Text Word] OR hyper polypharmacy[Text Word] OR polypharmacy[Text Word] OR poly-pharmacy[Text Word] OR poly pharmacy[Text Word] OR polytherapy[Text Word] OR poly-therapy[Text Word] OR poly therapy[Text Word] OR poly-medication[Text Word] OR polymedication[Text Word] OR poly medication[Text Word] OR multiple medication*[Text Word] OR multiple prescription*[Text Word] OR combination pharmacotherapy[Text Word]) | [9816](https://www.ncbi.nlm.nih.gov/pubmed/?cmd=HistorySearch&querykey=2) | 12:31:19 |
| [#1](https://www.ncbi.nlm.nih.gov/pubmed/advanced) | [Add](https://www.ncbi.nlm.nih.gov/pubmed/advanced) | Search Polypharmacy[MeSH Terms] | [3419](https://www.ncbi.nlm.nih.gov/pubmed/?cmd=HistorySearch&querykey=1) | 12:31:0 |

**Embase**

| No. | Query | Results |
| --- | --- | --- |
| #13 | **#11** NOT **#12** | **1863** |
| #12 | **#11** AND **'conference abstract'**/it | **591** |
| #11 | **#5** OR **#7** OR **#10** | **2454** |
| #10 | **#4** AND **#9** | **1828** |
| #9 | **child***:ti,ab OR **infant***:ti,ab OR **neonate***:ti,ab OR **toddler***:ti,ab OR **adolescent***:ti,ab OR **teen***:ti,ab OR **pediatric***:ti,ab OR **paediatric***:ti,ab OR **school**:ti,ab OR **boy***:ti,ab OR **girl***:ti,ab OR **baby**:ti,ab OR **babies**:ti,ab OR **newborn***:ti,ab OR **juvenile***:ti,ab OR **minors**:ti,ab | **2563695** |
| #7 | **#4** AND **#6** | **70** |
| #6 | **'hospitalized child'**/exp OR **'child health care'**/exp OR **'hospitalized adolescent'**/exp OR **'hospitalized infant'**/exp | **89500** |
| #5 | **#4** AND ([adolescent]/lim OR [child]/lim OR [infant]/lim OR [preschool]/lim OR [school]/lim) | **1785** |
| #4 | **#1** OR **#2** OR **#3** | **18226** |
| #3 | ((**prescribe*** OR **prescription***) NEAR/10 (**'multiple drug'** OR **'multiple drugs'**)):ti,ab | **130** |
| #2 | **hyperpolypharmacy**:ti,ab OR **'hyper polypharmacy'**:ti,ab OR **polypharmacy**:ti,ab OR **'poly pharmacy'**:ti,ab OR **polytherapy**:ti,ab OR **'poly therapy'**:ti,ab OR **polymedication**:ti,ab OR **'poly medication'**:ti,ab OR **'multiple medication'**:ti,ab OR **'multiple medications'**:ti,ab OR **'multiple prescription'**:ti,ab OR **'multiple prescriptions'**:ti,ab OR **'combination pharmacotherapy'**:ti,ab | **13358** |
| #1 | **'polypharmacy'**/exp | **11148** |

**EBSCO CINAHL**

| **#** | **Query** | **Limiters/Expanders** | **Last Run Via** | **Results** |
| --- | --- | --- | --- | --- |
| S10 | S5 OR S7 OR S9 | Search modes - Boolean/Phrase | Interface - EBSCOhost Research Databases  Search Screen - Advanced Search  Database - CINAHL with Full Text | 281 |
| S9 | S4 AND S8 | Search modes - Boolean/Phrase | Interface - EBSCOhost Research Databases  Search Screen - Advanced Search  Database - CINAHL with Full Text | 225 |
| S8 | child* or infant* or neonate* or toddler* or adolescent* or teen* or pediatric* or paediatric* or school or boy* or girl* or baby or babies or newborn* or juvenile* or minors | Search modes - Boolean/Phrase | Interface - EBSCOhost Research Databases  Search Screen - Advanced Search  Database - CINAHL with Full Text | 576,859 |
| S7 | S4 AND S6 | Search modes - Boolean/Phrase | Interface - EBSCOhost Research Databases  Search Screen - Advanced Search  Database - CINAHL with Full Text | 5 |
| S6 | (MH "Child, Hospitalized") OR (MH "Infant, Hospitalized") OR (MH "Adolescent, Hospitalized") | Search modes - Boolean/Phrase | Interface - EBSCOhost Research Databases  Search Screen - Advanced Search  Database - CINAHL with Full Text | 3,489 |
| S5 | S1 OR S2 OR S3 | Narrow by SubjectAge: - all child  Search modes - Boolean/Phrase | Interface - EBSCOhost Research Databases  Search Screen - Advanced Search  Database - CINAHL with Full Text | 208 |
| S4 | S1 OR S2 OR S3 | Search modes - Boolean/Phrase | Interface - EBSCOhost Research Databases  Search Screen - Advanced Search  Database - CINAHL with Full Text | 3,238 |
| S3 | ((prescribe* or prescription*) n10 (multiple drug*)) | Search modes - Boolean/Phrase | Interface - EBSCOhost Research Databases  Search Screen - Advanced Search  Database - CINAHL with Full Text | 17 |
| S2 | hyperpolypharmacy or hyper-polypharmacy or hyper polypharmacy or polypharmacy or poly-pharmacy or poly pharmacy or polytherapy or poly-therapy or poly therapy or poly-medication or polymedication or poly medication or multiple medication* or multiple prescription* or combination pharmacotherapy | Search modes - Boolean/Phrase | Interface - EBSCOhost Research Databases  Search Screen - Advanced Search  Database - CINAHL with Full Text | 3,228 |
| S1 | (MH "Polypharmacy") OR (MH "Polypharmacy (Saba CCC)") | Search modes - Boolean/Phrase | Interface - EBSCOhost Research Databases  Search Screen - Advanced Search  Database - CINAHL with Full Text | 2,072 |

**PsycINFO** <1806 to July Week 1 2017> Search Strategy:

--------------------------------------------------------------------------------

1 exp POLYPHARMACY/ (970)

2 ((prescribe* or prescription*) adj10 multiple drug*).ti,ab,id. (22)

3 (hyperpolypharmacy or hyper-polypharmacy or hyper polypharmacy or polypharmacy or poly-pharmacy or poly pharmacy or polytherapy or poly-therapy or poly therapy or poly-medication or polymedication or poly medication or multiple medication* or multiple prescription* or combination pharmacotherapy).ti,ab,id. (2477)

4 1 or 2 or 3 (2746)

5 limit 4 to (100 childhood <birth to age 12 yrs> or 200 adolescence <age 13 to 17 yrs>) (376)

6 (child* or infant* or neonate* or toddler* or adolescent* or teen* or pediatric* or paediatric* or school or boy* or girl* or baby or babies or newborn* or juvenile* or minors).ti,ab,id. (1007883)

7 4 and 6 (382)

8 5 or 7 (509)

**Web of Science Core Collection**

**Results: 1,037**

*(from Web of Science Core Collection)*

**TOPIC:**(("hyperpolypharmacy" or "hyper-polypharmacy" or "hyper polypharmacy" or "polypharmacy" or "poly-pharmacy" or "poly pharmacy" or "polytherapy" or "poly-therapy" or "poly therapy" or "multiple medication*" or "multiple prescription*" or "combination pharmacotherapy")) *AND* **TOPIC:** ((child* or infant* or neonate* or toddler* or adolescent* or teen* or pediatric* or paediatric* or school or boy* or girl* or baby or babies or newborn* or juvenile* or minors))

**Timespan:** All years. **Indexes:** SCI-EXPANDED, SSCI, A&HCI, CPCI-S, CPCI-SSH, BKCI-S, BKCI-SSH, ESCI, CCR-EXPANDED, IC.
