# Supplementary material for: Defining pediatric polypharmacy: A scoping review
Source: PLoS One. 2018 Nov 29;13(11):e0208047. doi: 10.1371/journal.pone.0208047 (PMC6264483; doi:10.1371/journal.pone.0208047)
Supplement: S1 Table — Research questions: Adverse events = drug-drug interactions, adverse reaction, adverse eventsMedication use = non-polypharmacy medication use questionsPrevalence = prevalence of polypharmacyOutcome of care = complications, death, prognostic measure, patient related outcomesDrug monitoring = drug levels, pharmacokinetics, pharmacodynamics.Threshold number of days were collapsed as follows: ≥1day category includes ≥1 day (51 studies) and ≥14 days (5)≥61 category includes ≥61 (5), ≥90 (3), ≥ 180 (4) and ≥365 (2)Sequential includes ≥1 year (6), 1 year (18), 2 years (7), and hospital stay (12). (DOCX) [file pone.0208047.s004.DOCX]

**S1 Table. Relationships between Research Questions and Definitions of Polypharmacy.**

| **Research Question** | **Medication Count** | **Duration** | **Number of Studies** | **Percent** |
| --- | --- | --- | --- | --- |
| Prognostic markers | >= 2 medications | Not reported | 74 | 17.87 |
| Prevalence | >= 2 medications | Not reported | 45 | 10.87 |
| Adverse events | >= 2 medications | Not reported | 36 | 8.70 |
| Other questions | >= 2 medications | Not reported | 29 | 7.00 |
| Drug monitoring | >= 2 medications | Not reported | 22 | 5.31 |
| Prevalence | >= 2 medications | 1 - 30 | 18 | 4.35 |
| Diagnostics | >= 2 medications | Not reported | 15 | 3.62 |
| Prevalence | >= 2 medications | Sequential | 14 | 3.38 |
| Medication use | >= 2 medications | Not reported | 9 | 2.17 |
| Prevalence | >= 2 medications | 31 - 60 | 8 | 1.93 |
| Adverse events | >= 2 medications | 1 - 30 | 8 | 1.93 |
| Prevalence | >= 2 medications | 061 -> 365 | 6 | 1.45 |
| Prevalence | Not reported | 1 - 30 | 6 | 1.45 |
| Medication use | >= 2 medications | Sequential | 6 | 1.45 |
| Medication use | Not reported | Sequential | 6 | 1.45 |
| Prognostic markers | >= 2 medications | 1 - 30 | 6 | 1.45 |
| Other questions | >= 2 medications | 1 - 30 | 5 | 1.21 |
| Other questions | >= 2 medications | Sequential | 5 | 1.21 |
| Prevalence | >= 3 medications | Sequential | 4 | 0.97 |
| Prevalence | Not reported | Sequential | 4 | 0.97 |
| Prevalence | Not reported | Not reported | 4 | 0.97 |
| Medication use | >= 2 medications | 1 - 30 | 4 | 0.97 |

1. Research questions:
   1. Adverse events=drug-drug interactions, adverse reaction, adverse events
   2. Medication use=non polypharmacy medication use questions
   3. Prevalence=prevalence of polypharmacy
   4. Outcome of care=complications, death, prognostic measure, patient related outcomes
   5. Drug monitoring=drug levels, pharmacokinetics, pharmacodynamics.
2. Some threshold number of days were collapsed as follows:
   1. >1day category includes >1 day (51 studies) and >14 days (5)
   2. >61 category includes >61 (5), >90 (3), > 180 (4) and >365 (2)
   3. Sequential includes >1 year (6), 1 year (18), 2 years (7), and hospital stay (12).
