# Supplementary material for: Defining pediatric polypharmacy: A scoping review
Source: PLoS One. 2018 Nov 29;13(11):e0208047. doi: 10.1371/journal.pone.0208047 (PMC6264483; doi:10.1371/journal.pone.0208047)
Supplement: S2 Table — AED = Antiepileptic drug AP = Antipsychotic LAS = Long-acting stimulants SSRI = Selective serotonin reuptake inhibitor CPT = Combined pharmacotherapy ADHD = Attention-deficit/hyperactivity disorder DP = Dispensed prescription WHO = World Health Organization VPA = Valproic acid CBZ = Carbamazepine PHT = Phenytoin CZP = Clonazepam SGA = Second generation antipsychotic ART = Artesunate AQ = Amodiaquine LCM = Lacosamide PDDI = Potential drug–drug interactions. (DOCX) [file pone.0208047.s005.DOCX]

**S2 Table. All Explicit Definitions of Pediatric Polypharmacy.**

| **Author** | **Disease Condition** | **Explicit Definition** |
| --- | --- | --- |
| **Studies that Specified Threshold Number of Medications and Overlapping Period** | | |
| **Carpay (1998)** | Epilepsy | • the concurrent use of 2 or more AEDs for more than 1 month |
| **Cho (2015)** | Epilepsy | • patients who received two or more AEDs on the same prescription date at least once |
| **Kanta (2014)** | Epilepsy | • two drugs were started simultaneously or second drug was added when first drug was not on maximum dose |
| **Baeza (2014)** | Psychiatry | • defining polypharmacy as the receipt of 2 or more AP (antipsychotic) medications concurrently for more than 60 days, with no gaps of more than 15 days in the treatment |
| **Bali (2015)** | Psychiatry | • concomitant use of long acting stimulants and atypical antipsychotics was defined as receipt of both medications together for at least 14 days |
| **Bhowmik (2013)** | Psychiatry | • polytherapy was defined as receiving medications with minimum 1 day overlap between prescriptions from two or three different therapeutic classes within a specific month |
| **Constantine (2010)** | Psychiatry | • antipsychotic polypharmacy was defined as the receipt of >=2 antipsychotic medications concurrently for >60 days, with no gaps in polypharmacy treatment >15 days |
| **Cornblatt (2007)** | Psychiatry | • 2 or more drugs taken at the same time |
| **Dosreis (2011)** | Psychiatry | • overlap of greater than or equal to 2 antipsychotics for more than 30 days. |
| **Fontanella (2009)** | Psychiatry | • the prescription of 3 or more medications from different drug classes at discharge. |
| **Geller (2010)** | Psychiatry | • polypharmacy was evident, in that 67.8% of subjects were taking medication from two or more medication classes concurrently during eight-year follow-up. |
| **Gyllenberg (2012)** | Psychiatry | • having purchased two psychotropic drugs from different drug classes during the same day. |
| **Kamble (2015)** | Psychiatry | • concurrent use or polypharmacy involving LAS and second-generation antipsychotics was defined as simultaneous receipt of both medications for at least 14 days |
| **Lee (2016)** | Psychiatry | • concurrent use of 2 or more antipsychotics for 90 days |
| **Lee (2016)** | Psychiatry | • concurrent use of 3 or more antipsychotics for 60 days |
| **Lee (2016)** | Psychiatry | • concurrent use of 3 or more antipsychotics for 90 days |
| **Logan (2015)** | Psychiatry | • the simultaneous use of two or more different classes of psychotropic medication for a period of at least 30 consecutive days at any time during the 2 year study period for each child |
| **Mandell (2008)** | Psychiatry | • concurrent use was coded when a child had prescriptions for >=3 medications in different classes overlapping for at least 30 days. |
| **Rubin (2009)** | Psychiatry | • concurrent use was coded when a child had prescriptions for >=3 medications in different classes overlapping for at least 30 days. |
| **Rubin (2012)** | Psychiatry | • concurrent use of 3 or more psychotropic medication classes for at least 30 days during the year |
| **Rushton (2001)** | Psychiatry | • patients were described as combination prescription recipients if they received both a stimulant and an SSRI during the same calendar year. |
| **Spencer (2013)** | Psychiatry | • polypharmacy was defined as at least 1 episode of multiclass polypharmacy. An episode of multiclass polypharmacy was defined as overlapping fills of medi- cations across >=2 classes for at least 30 days. |
| **Yoon (2012)** | Somatic | • combination therapy was defined as prescription claims for 2 drug classes on the same or within 1 day. |
| **Connor (1997)** | Multiple | • CPT (combined pharmacotherapy) was defined broadly as receiving two or more psychoactive agents at the same time |
| **Jameel (2012)** | Multiple | • polypharmacy: Nearly 20% of all the patients in our study were started on 2 or more psychotropic drugs simultaneously. |
| **Kalilani (2017)** | Multiple | • polytherapy was defined as the prescription of lacosamide concomitantly with another AED(s) with an overlap of at least 90 days. |
| **Martin (2003)** | Multiple | • multiple psychotropic pharmacotherapy was defined as having claims for prescriptions for medications in two or more different psychotropic drug classes during a seven-day period. |
| **Osunsanmi (2016)** | Multiple | • concurrent use of more than one ADHD medication for a continuous period of 6 months was referred to as cases on multiple medications. |
| **Schubart (2014)** | Multiple | • concurrent use defined as use of two or more medications overlapping for at least 60 days |
| **dosReis (2005)** | Multiple | • months of multiple use, which referred to the use of two or more different psychotropic classes within the same month. |
| **Allaire (2016)** | Not Reported | • having a prescription overlap of more than 30 days of two different second generation antipsychotics |
| **Feinstein (2015)** | Not Reported | • >=2 concurrent medications for at least 1day |
| **Feinstein (2015)** | Not Reported | • depth and duration: The cut point for high-depth was >= 5 concurrent medications, the cut point for high-duration was >= 31 days |
| **Hincapie-Castillo (2017)** | Not Reported | • overlap of greater than 45 days in the active periods of two or more psychotropic medications with different active ingredients |
| **Hovstadius (2009)** | Not Reported | • the prevalence of multiple medications was defined as the proportion of individuals who received five or more dispensed drugs during a 12-month period. |
| **Hovstadius (2010)** | Not Reported | • as a definition of excessive polypharmacy, we applied ten or more dispensed drugs (DP=10) for an individual during the study period |
| **Hovstadius (2010)** | Not Reported | • the prevalence of polypharmacy was defined as the proportion of individuals receiving five or more dispensed prescription drugs (DP=5) during a 3-month period. |
| **Sharma (2016)** | Not Reported | • the WHO standard for average number of drugs prescribed per patient encounter is 2.0. Rates higher than this standard are suggestive of polypharmacy. |
| **Zoega (2009)** | Not Reported | • concomitant drug use was defined as the dispensing of two or more different psychotropic chemical substances to a child on the same day at least once within the calendar year. |
| **Studies that Specified Threshold Number of Medications But Not Overlapping Period** | | |
| **Al-Qudah (1991)** | Epilepsy | • carbamazepine plus one or more AED |
| **Alexandre (2010)** | Epilepsy | • a combination of three or more AEDs |
| **Allarakhia (1996)** | Epilepsy | • VPA (valproic acid) plus 1 to 3 other antiepileptic drugs |
| **Cepelak (1998)** | Epilepsy | • VPA (valproic acid) + CBZ (carbamazepine) polytherapy |
| **Chakova (1998)** | Epilepsy | • a combined treatment with two or more AED |
| **Chen (2007)** | Epilepsy | • combinations of 2 or at most 3 drugs are usually prescribed for those unresponsive to monotherapy. |
| **Cloyd (1993)** | Epilepsy | • children in the polytherapy group received one or more other antiepileptic drugs. |
| **Coppola (2012)** | Epilepsy | • polytherapy (>=2 AEDs) |
| **Fong (2016)** | Epilepsy | • >1 AED |
| **Hamer (2012)** | Epilepsy | • the rate of polytherapy consisting of >2 AED |
| **Helal (2013)** | Epilepsy | • polypharmacy, the use of two or more anticonvulsants. |
| **Hernandez (2002)** | Epilepsy | • more than one antiepileptic drug |
| **Incecik (2014)** | Epilepsy | • two or three AEDs |
| **KarasalIhoGlu (2003)** | Epilepsy | • children who were using more than one AED were defined as receiving polypharmacy |
| **Kwong (1998)** | Epilepsy | • the use of polytherapy (two AED or more) |
| **Kwong (2016)** | Epilepsy | • polytherapy (2 or more antiepileptic drugs) |
| **Lagunju (2016)** | Epilepsy | • children on polytherapy, i.e.,>1 AED |
| **Larson (2012)** | Epilepsy | • current AED polytherapy: current AEDs >=2 |
| **Larson (2012)** | Epilepsy | • lifetime AED polytherapy: lifetime AEDs >=3 |
| **Lee (2015)** | Epilepsy | • polytherapy (two or more AEDs) |
| **Nettekoven (2008)** | Epilepsy | • two or more antiepileptic drugs |
| **Osama (2016)** | Epilepsy | • receiving more than one AED |
| **Poudel (2016)** | Epilepsy | • more than one antiepileptic drugs |
| **Racaru (2013)** | Epilepsy | • PT group (polytherapy treatment) for those having an association of two or three antiepileptic drugs. |
| **Reilly (2014)** | Epilepsy | • 2 AEDs or more |
| **Reilly (2014)** | Epilepsy | • current treatment (1 AED [monotherapy] vs 2 AEDs or more [polytherapy]) |
| **Sanchez (1986)** | Epilepsy | • polytherapy comprised carbamazepine plus phenobarbitone, valproate or phenytoin |
| **Schwabe (2001)** | Epilepsy | • topiramate and a second antiepileptic drug that was an enzyme inducing drug defined as phenobarbital, phenytoin, and carbamazepine |
| **Schwabe (2001)** | Epilepsy | • polytherapy with topiramate and a non–enzyme-inducing antiepileptic drug |
| **Selassie (2008)** | Epilepsy | • polytherapy of AEDs (defined as two or more AEDs) |
| **Sobaniec (2006)** | Epilepsy | • remaining 24 patients were treated with a heterogeneous combination of two or more of the following antiepileptic drugs |
| **Star (2014)** | Epilepsy | • more than one reported AED (i.e., reports listing AEDs in addition to valproic acid) |
| **Sugimoto (1996)** | Epilepsy | • standard-dose VPA plus phenytoin (PHT) polytherapy |
| **Sugimoto (1996)** | Epilepsy | • high-dose VPA plus PHT polytherapy |
| **Summers (1986)** | Epilepsy | • the prescribing of more than one anticonvulsant per patient |
| **Suzuki (1991)** | Epilepsy | • VPA was given with other antiepileptic drugs |
| **Talarska (2011)** | Epilepsy | • consisting of two or three AEDs |
| **Unay (2006)** | Epilepsy | • taking valproate plus carbamazepine therapy |
| **Verrotti (2004)** | Epilepsy | • 35 subjects received two or three drugs |
| **Verrotti (2012)** | Epilepsy | • 63 subjects had received combined drug therapy (two or three drugs) |
| **Wang (2016)** | Epilepsy | • a pharmacotherapy that prescribing more than two AEDs. |
| **Williams (1996)** | Epilepsy | • polytherapy consisted of two antiepileptic drugs with no child taking more than two drugs |
| **Yukawa (1991)** | Epilepsy | • patients are described as receiving polytherapy(A) if prescribed two anti-epileptic drugs including VPA. |
| **Yukawa (1991)** | Epilepsy | • polytherapy (B) describes the prescription of three or more anti-epileptic drugs including VPA. |
| **Yukawa (1992)** | Epilepsy | • patients are described as receiving polytherapy (A) if prescribed two anti-epileptic drugs including CBZ. |
| **Yukawa (1992)** | Epilepsy | • polytherapy (B) describes the prescription of three or more anti-epileptic drugs including CBZ. |
| **Yukawa (1992)** | Epilepsy | • polypharmacy (-VPA) refers to treatment involving more than one anti-epileptic drug with the exception of VPA. |
| **Yukawa (1992)** | Epilepsy | • polypharmacy (+VPA) refers to treatment involving more than one anti-epileptic drug including VPA. |
| **Yukawa (2001)** | Epilepsy | • polytherapy (A) if prescribed two antiepileptic drugs, including CZP. |
| **Yukawa (2001)** | Epilepsy | • polytherapy (B) describes the prescription of three or more antiepileptic drugs including CZP. |
| **Costa (2017)** | Psychiatry | • using three or more medications at the time of collection in the medical record |
| **Dean (2006)** | Psychiatry | • polypharmacy (>1 concurrent drug) |
| **Essock (2009)** | Psychiatry | • >=3 psychotropics for children |
| **Fegert (2006)** | Psychiatry | • comedication during the year, measured either as more than one antidepressant subclass or more than one antidepressant drug |
| **Fontanella (2009)** | Psychiatry | • 3 or more different drug combinations |
| **Griffith (2010)** | Psychiatry | • taking two or more psychotropic medications |
| **Gyllenberg (2012)** | Psychiatry | • the concomitant use of two psychotropic drugs from different drug classes. |
| **Hilt (2014)** | Psychiatry | • in order to avoid sparse observations in categories of polypharmacy defined by use of a drug class, we redefined polypharmacy to reflect use of more than one medication. |
| **Huefner (2017)** | Psychiatry | • treatment using two or more psychotropic medications |
| **Jerrell (2008)** | Psychiatry | • 1756 patients (42.3%) were prescribed more than one SGA or SGA and a conventional agent during the follow-up period (either sequentially or as concomitant pharmacotherapy). |
| **Lake (2014)** | Psychiatry | • the concurrent use of two or more psychotropic medications in the same individual, either within or between therapeutic classes. |
| **Lekhwani (2004)** | Psychiatry | • >/=2 medications |
| **Logan (2012)** | Psychiatry | • number of different psychotropic classes prescribed during the study period was counted, and two dichotomous variables indicated the presence of claims from (i) more than one, and (ii) three or more different psychotropic classes. |
| **Mattison (2014)** | Psychiatry | • polypharmacy with two or more psychotropic medications |
| **McIntyre (2009)** | Psychiatry | • >=2 psychotrophic medications |
| **Morrato (2007)** | Psychiatry | • the index antipsychotic drug was classified as conventional, atypical, or, if >1 antipsychotic drug was dispensed on the index date, multiple. |
| **Murray (2014)** | Psychiatry | • given prescriptions for two or more drugs |
| **Pappadopulos (2002)** | Psychiatry | • the administration of two or more psychotropic medications (polypharmacy) is also an area of concern |
| **Raghavan (2008)** | Psychiatry | • use of two or more concurrent psychotropic medications. |
| **Trinczek (2016)** | Psychiatry | • antipsychotic polypharmacy (continuous parallel prescription of >=2 antipsychotic substances) |
| **Adams (2001)** | Somatic | • 3 or more controller dispensings |
| **Adams (2001)** | Somatic | • 5 or more controllers dispensing |
| **Brinkman (2008)** | Somatic | • use of more than one medication |
| **Dodoo (2009)** | Somatic | • combination therapy (ART-AQ: artesunate plus amodiaquine) |
| **Dodoo (2009)** | Somatic | • combination therapy (other ACT: patients taking an artemisinin derivative in combination with a non-artemisinin-containing anti-malarial) |
| **Lass (2011)** | Somatic | • altogether 63% (n=73) of preterm and 38% (n=94) of term neonates received more than three drugs. |
| **Manjhi (2016)** | Somatic | • polypharmacy (>3 drugs) |
| **Okoro (1995)** | Somatic | • multiple drug use, a situation in which about 57% of all drug-users used between 2 and 4 or more drugs. |
| **Rashed (2012)** | Somatic | • average number of prescriptions per patient (in group: <5, =5) |
| **Yoon (2012)** | Somatic | • a repetitive pattern of prescription claims for 2 drug classes |
| **Zeng (2017)** | Somatic | • more than one antibiotic was used |
| **Betts (2014)** | Multiple | • for two stimulants to qualify as concomitant usage, the new stimulant had to be a different class than the index stimulant. |
| **Blader (2006)** | Multiple | • received treatment with 3 or more medications |
| **Brauner (2016)** | Multiple | • prescription of 2 or more psychopharmacological drugs per patient |
| **Comer (2010)** | Multiple | • The term “polypharmacy” refers to the “administration of many drugs together” or “the administration of excessive medication." Although no specific number of concurrently prescribed medications can unequivocally define polypharmacy, in the present study we were interested in trends in the concurrent prescription of two or more psychotropic drugs from across medication classes. |
| **Comer (2010)** | Multiple | • patient was prescribed two or more psychotropic agents from across different psychotropic medication classes |
| **Connor (1997)** | Multiple | • polypharmacy is the intentional and concomitant use of more than two medications to treat either a patient with more than one pathophysiologically distinct illness or a patient with a single disorder. |
| **Duffy (2005)** | Multiple | • the concurrent use of two or more psychopharmacologic medications for the treatment of psychiatric disorder(s) in children and adolescents |
| **Duffy (2005)** | Multiple | • concurrent use of two or more combinations of psychopharmacologic agents from the same medication class, or drug combinations from different medication classes |
| **Esbensen (2009)** | Multiple | • more than one psychotropic drug class |
| **Fontanella (2014)** | Multiple | • involved three or more medications from the same or different drug classes |
| **Fontanella (2014)** | Multiple | • involved three or more medications from different drug classes |
| **Gururaj (2003)** | Multiple | • poly therapy with more than 1 antiepileptic drug |
| **Hobbs (2014)** | Multiple | • receiving medications from two or more of the categories listed above |
| **Hong (2010)** | Multiple | • the simultaneous use of two or more scheduled antipsychotics |
| **Kalilani (2017)** | Multiple | • the use of concomitant AED(s) was defined as the presence of at least one prescription claim of another AED(s) other than lacosamide, with an overlap in the supply days with LCM prescriptions |
| **Knezevic-Pogancev (2011)** | Multiple | • more than one AED |
| **Kowatch (2013)** | Multiple | • polypharmacy was operationally defined as taking two or more psychiatric medications concurrently. |
| **Kulak (2003)** | Multiple | • two AEDs or more |
| **Malerba (2010)** | Multiple | • a combination of two of more AEDs |
| **Olashore (2017)** | Multiple | • when two or more psychotropic medications (i.e., antipsychotics, antidepressants, anticholinergics, stimulants, mood stabilizers, etc.) were used at the same treatment period |
| **Olashore (2017)** | Multiple | • the prescription of two or more psychiatric medications concurrently to a patient |
| **Olashore (2017)** | Multiple | • polypharmacy, defined as the prescription of two or more medications concurrently to a patient |
| **Olashore (2017)** | Multiple | • a patient is considered to receive multi-drug treatment when more than two medications were given at the same time. |
| **Procyshyn (2014)** | Multiple | • patients prescribed with a combination of 2 antipsychotics |
| **Reilly (2015)** | Multiple | • two AEDs or more |
| **Saldana (2014)** | Multiple | • the use of more than one antipsychotic concomitantly |
| **Sullivan (2015)** | Multiple | • polypharmacy->simultaneously using two or more medications, e.g., Effexor and Lexapro |
| **Sullivan (2015)** | Multiple | • multi-class pharmacotherapy->simultaneously using two or more medications from at least two different medication classes, e.g., Ritalin and Prozac |
| **Zito (2008)** | Multiple | • concomitant use of >=2 drugs within the same class |
| **Zito (2008)** | Multiple | • >=3 psychotropic medication classes concomitantly |
| **Zito (2008)** | Multiple | • receive >=4 psychotropic medication classes concomitantly |
| **Allaire (2016)** | Not Reported | • two or more psychotropic drugs |
| **Bergendal (2015)** | Not Reported | • use of two or more antipsychotic drugs and other psychotropic drugs |
| **Bhatara (2004)** | Not Reported | • >1 psychotropic prescribed |
| **Brenner (2014)** | Not Reported | • youth taking two or more psychotropic medications. |
| **Chen (2011)** | Not Reported | • concurrent use of =2 psychotropic medications for the treatment of psychiatric disorders among children and adolescents. |
| **Dai (2016)** | Not Reported | • five or more drugs as threshold for polypharmacy exposed to at least one PDDI (potential drug–drug interactions) |
| **Feinstein (2015)** | Not Reported | • depth: low [2-4 concurrent medications], medium [5-9], and high [>=10]) based on a patient's maximum daily depth of polypharmacy during the study period |
| **Hug (1991)** | Not Reported | • two-drug regimens |
| **Knopf (2010)** | Not Reported | • polypharmacy (>=2) |
| **Kurian (2016)** | Not Reported | • receive greater than or equal to 4 medications |
| **Lemer (2009)** | Not Reported | • more than 1 medication |
| **Martinbiancho (2007)** | Not Reported | • the concomitant and extended utilization of two or more drugs in a treatment, either due to the patient’s pathological condition or the need for action or effect complementation, is known as polypharmacy. |
| **Pandey (2010)** | Not Reported | • at least two or more prescribed drugs. |
| **Studies that Did Not Specify Threshold Number of Medications But Specified Overlapping Period** | | |
| **Bhowmik (2014)** | Psychiatry | • receiving medications from different therapeutic classes within 30 days of the depression diagnosis and with a minimum of 14 day overlap between the prescriptions |
| **Essock (2009)** | Psychiatry | • a period of simultaneous prescribing of psychotropic medications for longer than 90 days, both within and across drug class (antipsychotics, antidepressants, sedative-hypnotics, nonantipsychotic mood stabilizers, and stimulants) |
| **Morrato (2007)** | Psychiatry | • antipsychotic polypharmacy was defined as initiation of multiple antipsychotic medications or at least 60 consecutive days of concomitant antipsychotic medication overlapping the index antipsychotic prescription at any time during the 365 days after the index drug claim. |
| **Wonodi (2007)** | Psychiatry | • concomitant conventional and atypical antipsychotic treatment for 6 months or more |
| **Wonodi (2007)** | Psychiatry | • chronically treated with atypical anti- psychotics but had received concomitant treatment with conventional antipsychotics, ranging from 1 to 172 days |
| **Betts (2014)** | Multiple | • for a medication fill to qualify as concomitant usage, the medication had to have been filled in 2009 and have at least 30 days of supply overlap with the index stimulant, and there had to be at least one primary diagnosis of ADHD recorded prior to the end of medication overlap. |
| **Chen (2011)** | Not Reported | • the prevalence of combination treatments was defined as receiving >=14, >=30, >=60, and >=90 consecutive days of overlapping psychotropic prescription fills. |
| **Faber (2005)** | Not Reported | • considering another prescription as co-medication only when the other psychotropic drug was dispensed in the same week as a stimulant prescription |
| **Hovstadius (2009)** | Not Reported | • the administration of many drugs at the same time or the administration of an excessive number of drugs |
| **Vallano (2004)** | Not Reported | • average number of medicines per encounter (N drugs). |
| **Studies that Neither Specified Threshold Number of Medications Nor Specified Overlapping Period** | | |
| **Chiba (1985)** | Epilepsy | • receiving VPA concurrently with other AEDs |
| **Chung (1997)** | Epilepsy | • VPA (valproic acid) plus other antiepileptic drugs |
| **Eldeen (2012)** | Epilepsy | • subgroup (B), receiving polytherapy treatment (combined treatment with valproic acid (VPA) and carbamazepine (CBZ)) |
| **Farhat (2002)** | Epilepsy | • on multiple AED therapy |
| **Furlanut (1985)** | Epilepsy | • polytherapy (CBZ associated with one or more of the following drugs: phenobarbital, primidone, phenytoin, ethosuximide) |
| **Kanemura (2015)** | Epilepsy | • group B, combination therapy of VPA with other conventional AED |
| **Kanemura (2015)** | Epilepsy | • group C, new AED administration after VPA therapy |
| **Liu (1994)** | Epilepsy | • carbamazepine plus other antiepileptics |
| **Mikati (2007)** | Epilepsy | • VPA with other AEDs |
| **Nakajima (2011)** | Epilepsy | • VPA combined with other anticonvulsants |
| **Ohtahara (2004)** | Epilepsy | • number of AEDs added to zonisamide |
| **Udani (1993)** | Epilepsy | • normal dose polytherapy- where multiple drugs were used, the doses being within accepted range |
| **Udani (1993)** | Epilepsy | • high dose polytherapy -where multiple drugs were used, of which at least 1 was in high doses |
| **Wang (1993)** | Epilepsy | • VPA in combination with other anticonvulsants |
| **Madden (2017)** | Psychiatry | • the total number of major classes dispensed |
| **Safer (1997)** | Psychiatry | • the recording of multiple concurrent psychotropic medication prescriptions (polypharmacy) |
| **Duffy (2005)** | Multiple | • concomitant pharmacotherapy could be the combination of psychopharmacologic agents from the same medication class, or could include drug combinations from different medication classes |
| **Liu (1994)** | Multiple | • receiving CBZ polytherdpy by taking CBZ and valproic acid (VPA) or CBZ plus other antiepileptic drugs (AEDs). |
| **Patel (2017)** | Multiple | • the proportion prescribed other psychotropic prescriptions in addition to ADHD medication |
| **Procyshyn (2014)** | Multiple | • the concomitant use of other psychotropic medications with antipsychotics. |
| **Sadahiro (1985)** | Multiple | • phenobarbital combined with other antiepileptic drugs |
| **Sadahiro (1985)** | Multiple | • valproic acid combined with other antiepileptic drugs |
| **Sarkar (2013)** | Multiple | • the concurrent use of multiple psychoactive medications in a single patient |
| **Schubart (2014)** | Multiple | • concurrent use of multiple medications within any single class |
| **Schubart (2014)** | Multiple | • concurrent use of psychotropic medications in different classes of medication |
| **Feinstein (2015)** | Not Reported | • polypharmacy, defined as the concurrent use of multiple medications |
| **Gilat (2011)** | Not Reported | • the use of multiple concurrent medications (polypharmacy) |
| **Olfson (2002)** | Not Reported | • frequency with which children used a medication from more than one psychotropic group |
| **Pandey (2010)** | Not Reported | • polypharmacy, as suggested by a high average number of drugs/encounter, and in high majority, 1345 (97.7%) prescriptions |

AED = Antiepileptic drug

AP = Antipsychotic

LAS = Long-acting stimulants

SSRI = Selective serotonin reuptake inhibitor

CPT = Combined pharmacotherapy

ADHD = Attention-deficit/hyperactivity disorder

DP = Dispensed prescription

WHO = World Health Organization

VPA = Valproic acid

CBZ = Carbamazepine

PHT = Phenytoin

CZP = Clonazepam

SGA = Second generation antipsychotic

ART = Artesunate

AQ = Amodiaquine

LCM = Lacosamide

PDDI = Potential drug–drug interactions
